# A surrogate marker for very early-stage tau pathology is detectable by molecular magnetic resonance imaging

**DOI:** 10.7150/thno.72258

**Published:** 2022-07-18

**Authors:** Parag Parekh, Qingshan Mu, Andrew Badachhape, Rohan Bhavane, Mayank Srivastava, Laxman Devkota, Xianwei Sun, Prajwal Bhandari, Jason L. Eriksen, Eric Tanifum, Ketan Ghaghada, Ananth Annapragada

**Affiliations:** 1 Baylor College of Medicine, Houston, TX, USA; 2 Texas Children's Hospital, Houston, TX, USA; 3 College of Pharmacy, University of Houston, Houston, TX, USA; 4 Texas Children's Hospital/Baylor College of Medicine, Houston, TX, USA

**Keywords:** Tau, Alzheimer's disease, Hyperphosphorylation, Magnetic Resonance Imaging (MRI), Thioaptamer, Withaferin A, Nanoparticle, Molecular Imaging

## Abstract

The abnormal phosphorylation of tau is a necessary precursor to the formation of tau fibrils, a marker of Alzheimer's disease. We hypothesize that hyperphosphorylative conditions may result in unique cell surface markers. We identify and demonstrate the utility of such surrogate markers to identify the hyperphosphorylative state.

**Methods:** Cell SELEX was used to identify novel thioaptamers specifically binding hyperphosphorylative cells. Cell surface vimentin was identified as a potential binding target of the aptamer. Novel molecular magnetic resonance imaging (M-MRI) probes using these aptamers and a small molecule ligand to vimentin were used for *in vivo* detection of this pre-pathological state.

**Results:** In a mouse model of pathological tau, we demonstrated *in vivo* visualization of the hyperphosphorylative state by M-MRI, enabling the identification at a pre-pathological stage of mice that develop frank tau pathology several months later. *In vivo* visualization of the hyperphosphorylative state by M-MRI was further validated in a second mouse model (APP/PS1) of Alzheimer's disease again identifying the mutants at a pre-pathological stage.

**Conclusions:** M-MRI of the hyperphosphorylative state identifies future tau pathology and could enable extremely early-stage diagnosis of Alzheimer's disease, at a pre-patholgical stage.

## Introduction

The microtubule-associated protein tau, coded for by the *MAPT* gene, is abundant in the brain and is present in neurons, glia, and other cell types. Tau expressed in six isoforms has a vast array of post-translational modifications, including glycosylation, glycation, nitration, ubiquitination and more than 80 potential phosphorylation sites expanding the complexity of its role in health and disease 1,2. A definitive feature of many neurodegenerative diseases, including Alzheimer's disease (AD), frontotemporal lobar degeneration (FTLD), and Parkinson's disease (PD) is the presence of intracellular aggregated filamentous tau (collectively termed “tauopathies”) 3-6. The transition from physiological soluble tau to insoluble tau is primarily associated with changes in its phosphorylation state leading to oligomerization 7. Tau fibrils known as paired helical fragments (PHF) then form characteristic neurofibrillary tangles (NFT). Tau aggregates are also capable of propagating into healthy cell, inducing tau misfolding, aggregation, and neuro-toxicity 8,9. Studies of intercellular propagation demonstrate passage through an extracellular phase that progresses throughout the brain 10,11.

The National Institute on Aging-Alzheimer's Association (NIA-AA) Research Framework identifies extracellular deposits of amyloid beta (A), presence of intraneuronal hyperphosphorylated tau (T) and markers of neurodegeneration or neuronal injury (N) as characteristics of AD. Each biomarker is scored either positive or negative 12. To be on the AD continuum, A+ (Amyloid positive) is required, while a positive diagnosis of AD requires A+ and T+. Biomarker detection can be by positron emission tomography (PET) imaging of amyloid and tau, cerebrospinal fluid (CSF) detection of reduced Aβ_42_, and/or high Aβ_40_/Aβ_42_ ratio, high phosphorylated tau and total tau, and neuronal injury or degeneration as shown by structural brain magnetic resonance imaging (MRI) 13. Tracking of brain pathology in longitudinal studies suggests that tau pathology may precede the accumulation of Aβ, but is undetectable based on current biomarker detection threshold levels, and is amplified catastrophically by independent Aβ deposition 14-16. The ATN research framework-based diagnosis of AD is therefore limited by tau pathology detection.

The initiation of tau pathology is marked by abnormal phosphorylation of tau 17,18. We hypothesize that hyperphosphorylative conditions in neurons, consistent with an altered balance of kinase-phosphatase activity causing elevated levels of hyperphosphorylated tau species, result in unique surface markers 19-21. The utility of such a surface-marker lies in the fact that an imaging agent can bind it without needing to penetrate the cell membrane, a limitation that currently hinders tau-PET agents. Reverse phase protein array (RPPA) analysis of a cell-based model of tau hyperphosphorylation identified a large number of proteins that were either up- or down-regulated by the onset of the hyperphosphorylative state. Using an iterative Cell-SELEX process we identified DNA thioaptamers that specifically bound such cells 22,23. High T1 relaxivity PEGylated liposomes bearing macrocyclic Gd-chelates 24,25 (Gd-Lips) were modified to present thioaptamers on their surface, thus enabling targeting of the particles to the surface of hyperphosphorylative cells for M-MRI. We sought the binding targets of the thioaptamers, and identified vimentin, a normally intracellular protein that is specifically expressed on the surface of cells under hyperphosphorylative conditions, as a possible cell surface marker of pathological hyperphosphorylation found in AD. We found cell-surface vimentin at elevated levels on SH-SY5Y and ReN-VM cells in the hyperphosphorylative state that also showed elevated phosphorylated Tau (pTau) levels, and that such cells were also specifically bound by the lead candidate aptamers. In 2-month-old P301S transgenic mice, we found elevated vimentin levels in cells of the hippocampus that also had elevated pTau levels, but non-transgenic sibling mice (born in the same litter) did not exhibit either elevated pTau or vimentin. We then conjugated Withaferin A, a small molecule vimentin ligand, to Gd-containing liposomes (Gd-Lips). When injected intravenously in P301S mice at 2 months of age, both the thioaptamer targeted Gd-Lips and the Withaferin targeted Gd-Lips produced signal enhancement on MRI in the brains of transgenic mice but not in the brains of non-transgenic siblings. Untargeted Gd-Lips did not show any signal enhancement in either group of mice. Practically 100% of the P301S mice go on to develop frank tau pathology at 8 months of age or later. The targeted Gd lips therefore serve as an M-MRI agent that can identify the development of future tau pathology in mice in a pre-pathological state.

## Results

### Validation of cell surface changes in hyperphosphorylative conditions

**Figure 1:**
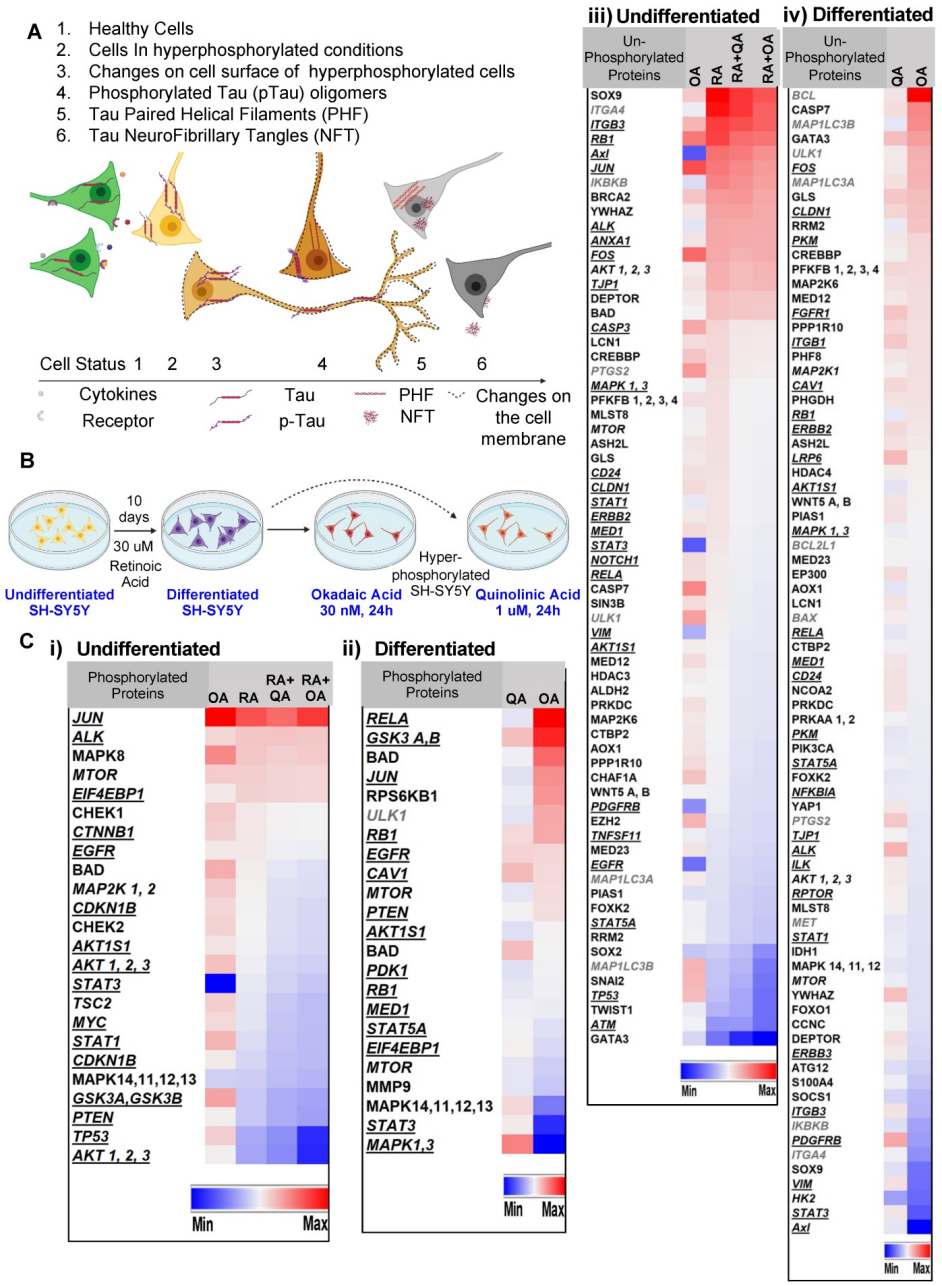
** Hyperphosphorylation and cell surface changes in a cell of neuronal phenotype. (A)** We hypothesize that cell surface changes occur concurrently with tau hyperphosphorylation, a precursor to tau fibrillation. Such surface changes may constitute a surrogate marker for early detection of AD. **(B)** SH-SY5Y cells differentiated to a neuronal phenotype by treatment with retinoic acid (RA) and treated with either okadaic acid (OA) or quinolinic acid (QA) are used to model hyperphosphorylative neurons. **(C)** Reverse phase protein array analysis of undifferentiated cells, RA treated and hyperphosphorylative cells (OA or QA treated). Heat map depicts statistically significant (p<0.05) changes in total protein expression from the reference condition. Proteins are classified as cell-membrane (bold italic underlined) peripheral-membrane proteins (bold italic not underlined) and single-pass and multipass membrane proteins (italic) and not membrane associated (bold). Changes due to phosphorylation treatments with OA and QA on i) undifferentiated and ii) differentiated SH-SY5Y cells. Changes in un-phosphorylated proteins on iii) undifferentiated and iv) differentiated SH-SY5Y cells.

SH-SY5Y cells treated with retinoic acid (RA)** (**as depicted in Figure** 1 A-B)** underwent differentiation to a neuronal phenotype marked by morphological changes including the lengthening of the cell body and the development or neurite-like processes. Cells treated with the membrane-permeating neurotoxin okadaic acid 26 (OA) were rendered hyperphosphorylative, demonstrating an increase in pTau S202/T205 and ptau S396 (Supplementary Figure** S1**). In parallel experiments, the excitotoxin quinolinic acid 27 (QA) was used to induce hyperphosphorylation.

Reverse-phase protein array (RPPA) analysis conducted on lysates of both RA-treated and untreated SH-SY5Y cells rendered hyperphosphorylative by either OA (30 nM, 24 h) or QA (1 µM, 24 h) demonstrated marked changes in hyperphosphorylative cells. We identified 98 proteins and 36 phosphorylated proteins that showed significant changes in expression levels (Figure** 1C**). Uniprot 28 protein association showed 44 cell-membrane associated proteins, 10 peripheral membrane and 12 single-pass membrane proteins were significantly altered under hyperphosphorylative conditions.

### Screening for aptamers that bind cells in hyperphosphorylative state

**Figure 2:**
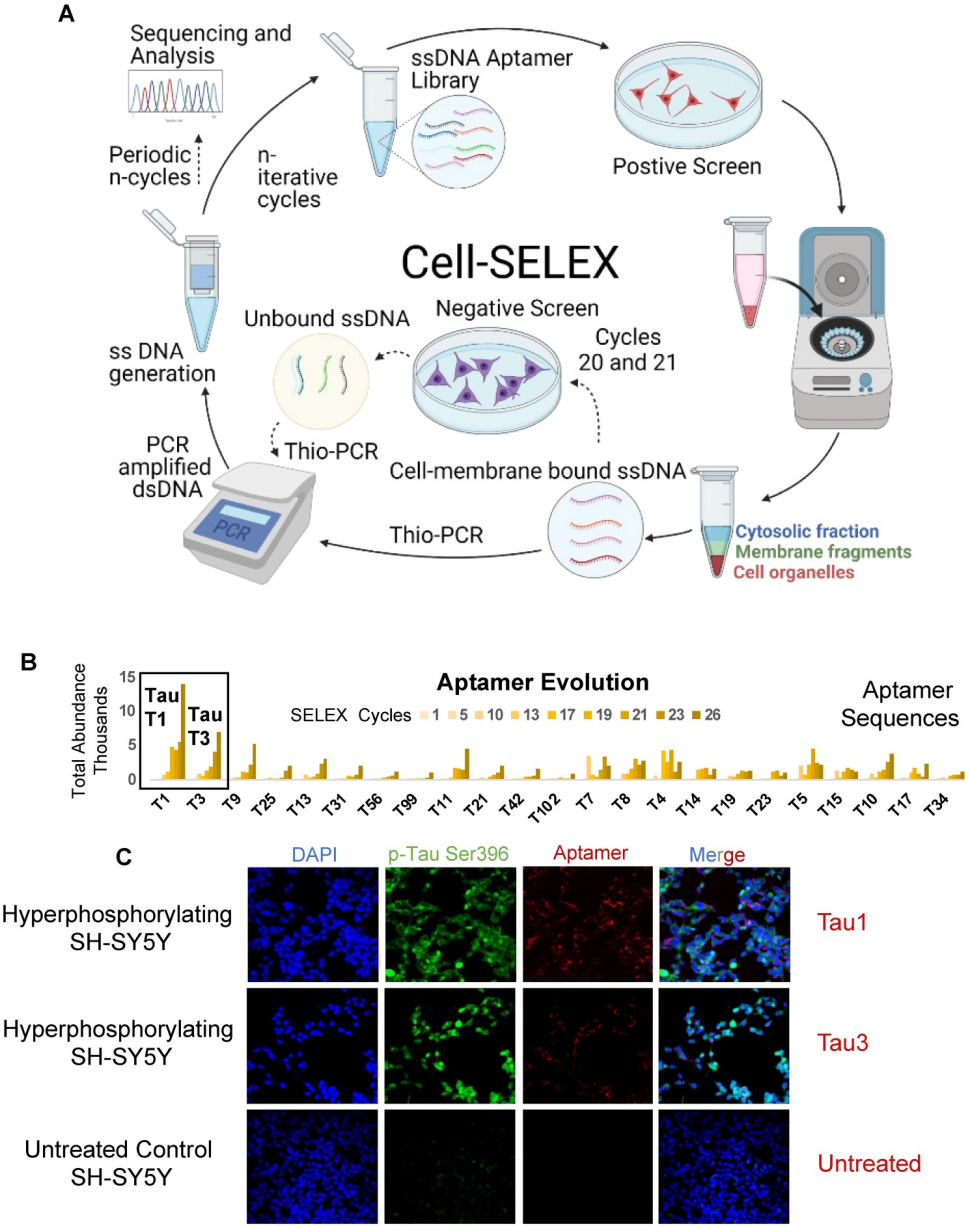
** Cell-SELEX to identify biomarkers onset of AD.** The cell-SELEX methodology modified to capture membrane binding aptamers was used to identify aptamers that specifically bind hyperphosphorylative neurons. Okadaic acid treated differentiated SH-SY5Y cells were used as a surrogate for hyperphosphorylative neurons to screen DNA aptamers that specifically recognize the differences between the surfaces of treated and untreated cells. **(A)** Pictorial representation of the cell-SELEX process. **(B)** Abundance of the top 23 sequences from SELEX cycle 1 - 26. Note that the fractions are low until about cycle 10, when they increase sharply. Note that the abundance of Tau 1, Tau 3 continually increase with increasing cycle number. **(C)** Hyperphosphorylated SH-SY5Y cells stained with 50nM Cy5 labelled Tau1, Tau3 aptamer for 2h, compared to an untreated control (lower row), imaged with a 40X objective.

Aptamer screening was performed using a modified cell-SELEX methodology on differentiated SH-SY5Y cells rendered hyperphosphorylative by okadaic acid (Figure **2A**). A total of 26 cell SELEX cycles were performed. To remove thioaptamers that bound cell surface molecules not specific to the hyperphosphorylative state, a negative selection was introduced at cycles 12 and 13 using differentiated, non hyperphosphorylative cells (i.e. without OA treatment). Anticipating that selected thioaptamers would be systemically delivered as nanoparticle imaging agents, the primary toxicity of which is driven by hepatocyte uptake, we conducted another round of negative selection at cycles 20 and 21 using a hepatocyte cell-line THLE-3 to remove thioaptamers that exhibited enhanced uptake by hepatocytes.

### Tau1 and Tau3 aptamers specifically bind hyperphosphorylative cells

**Figure 3:**
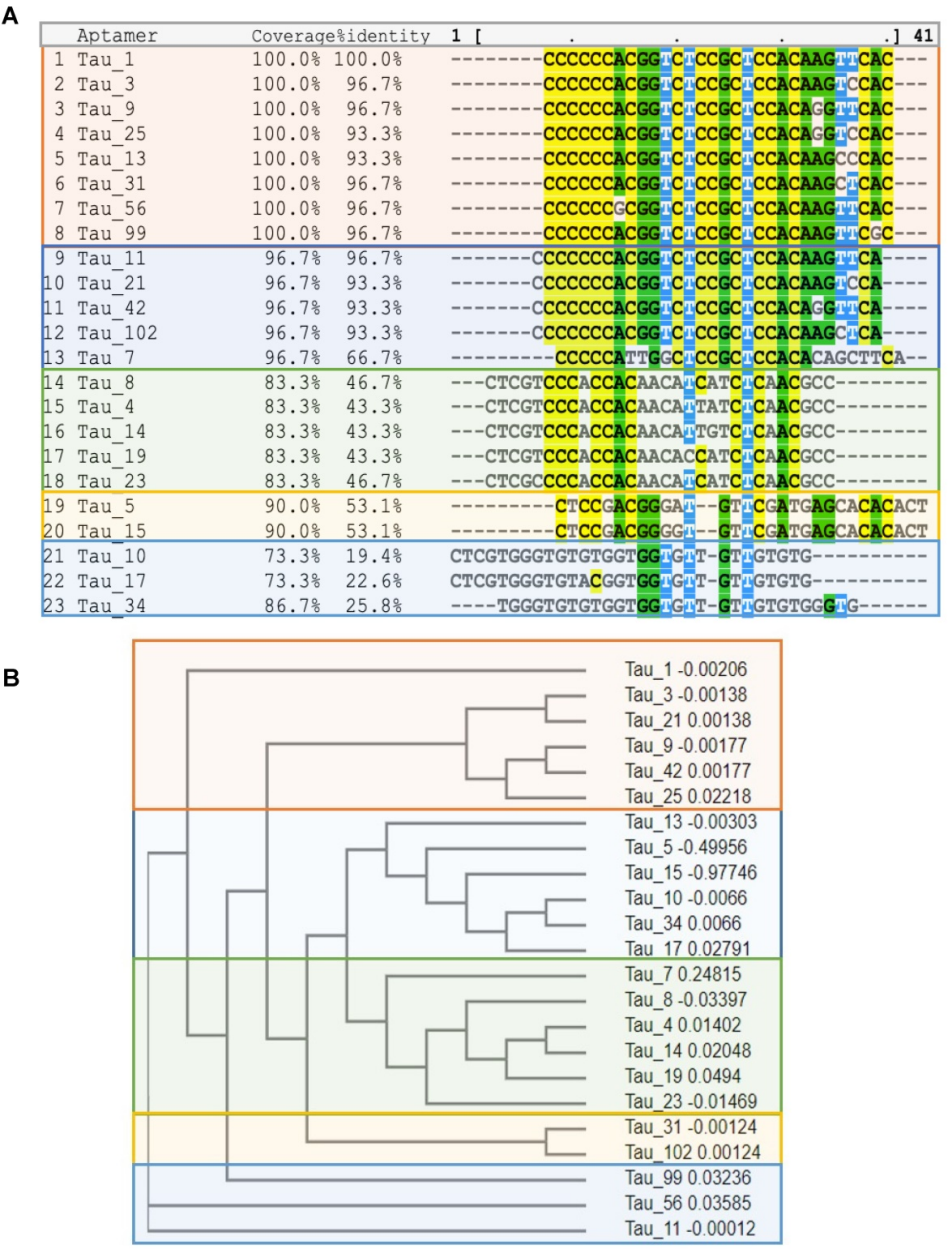
** (A)** Multiple sequence alignment of the top 23 aptamer sequences by MAFFT. Note the existence of five families. **(B)** Cladogram showing relationship between the Tau aptamer sequences and the aptamer families.

Sequencing of all the selected pools using the Ion Torrent sequencing platform 29 revealed the evolution of families of DNA sequences, with enrichment particularly evident after 10 rounds of SELEX. Negative selection eliminated certain sequences that were not specific to the hyperphosphorylative state or have a propensity for hepatocyte uptake. However, the relative abundance of key sequences increased steadily throughout the whole process. The 23 most abundant sequences at round 26 were identified and their abundance throughout the SELEX process as calculated using AptaAligner 30 is shown in Figure **2B**. Tau1 was the most prevalent at cycle 26, representing 20.6% of thioaptamers present. A single base difference from this sequence, Tau3, was the second most represented sequence (10.4%). Hyperphosphorylative SH-SY5Y cells were bound by both Cy5-labeled Tau1and Tau3 aptamers (Figure **2C**). The secondary structure of the aptamers Tau1 and Tau3 calculated using mfold 31 is shown in Supplementary Figure **S2**. The sequences present at the final round were grouped by hierarchical clustering and sequence homology using the multiple sequence alignment code MAFFT 32 and yielded five distinct families (Figure **3A**) presented as a cladogram (using the Clustal Omega algorithm 33) showing the common structural root of these five thioaptamer families (Figure **3B**). The apparent equilibrium dissociation constants (Kd_app_) were measured by serial dilution of aptamer solutions with target hyperphosphorylated, and non-target differentiated and undifferentiated SH-SY5Y cells. The affinity of these aptamers was also tested with another immortal neural progenitor stem cell line ReN-VM 34 in hyperphosphorylative and non-hyperphosphorylative conditions. The Kd_app_ for Tau1 and Tau3 to hyperphosphorylated SH SY5Y cells are 0.167 ± 0.01 nM and 0.194 ± 0.03 nM; and for the ReN-VM cells 318.15 ± 46.20 nM and 234.24 ± 38.60 nM respectively (Supplementary Figure** S3**).

### Binding target identification

**Figure 4:**
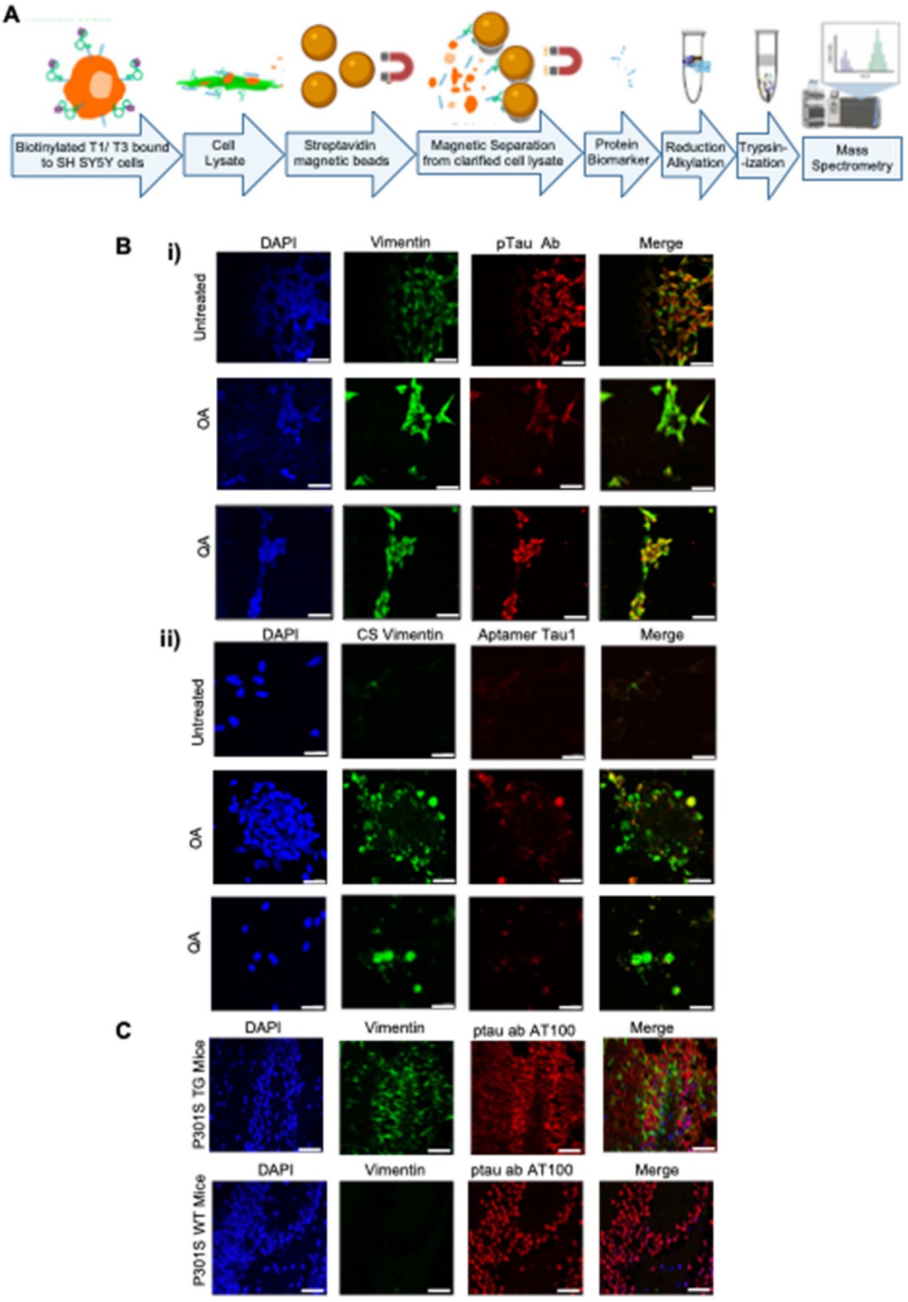
** Potential binding target of the aptamer. (A)** Tryptic digest of aptamer bound cell membrane analyzed by LC/MS/MS. The raw data files were processed and searched against the SwissProt 2012 01 (Human) database. Vimentin was identified as possible target. **(B)** Presence of the aptamer binding target on SH SY5Y cells; undifferentiated and hyperphosphorylative OA (24 h, 30 nM) and QA (24 h, 100 nM) treated; i) co-stained with Vimentin (D21H3) and ptau (AT100) antibodies ii) cell surface (CS) vimentin (Clone 84-1) antibody and aptamer T1 (50 nM); nuclei counterstained with DAPI. **(C)** Expression of Vimentin in P301S TG and WT frozen mouse tissue sections stained with Vimentin (SP20) and ptau (AT100) antibodies and DAPI stained nuclei.

To characterize the binding target of the aptamers, we performed a bead-based pulldown assay, followed by mass-spectrometry (Figure **4A**). We performed the assay for both Tau1 and Tau3, aptamers. A ranking of the abundance scores for identified proteins revealed Vimentin as a possible binding target among many others (Supplementary **Table S1**). SH-SY5Y cells under undifferentiated and differentiated hyperphosphorylative conditions showed increasing levels of vimentin (Figure **4Bi**) further suggesting it as a potential target of aptamers Tau1 and Tau3 specific to the hyperphosphorylative state. Tau1 aptamers also co-locate with an anti-CSV antibody when exposed to hyperphosphorylative cells (Figure **4Bii**). Vimentin expression was higher in brains of P301S transgenic mice when compared to WT mice (Figure **4C**).

### TauX nanoparticles for magnetic resonance imaging

**Figure 5:**
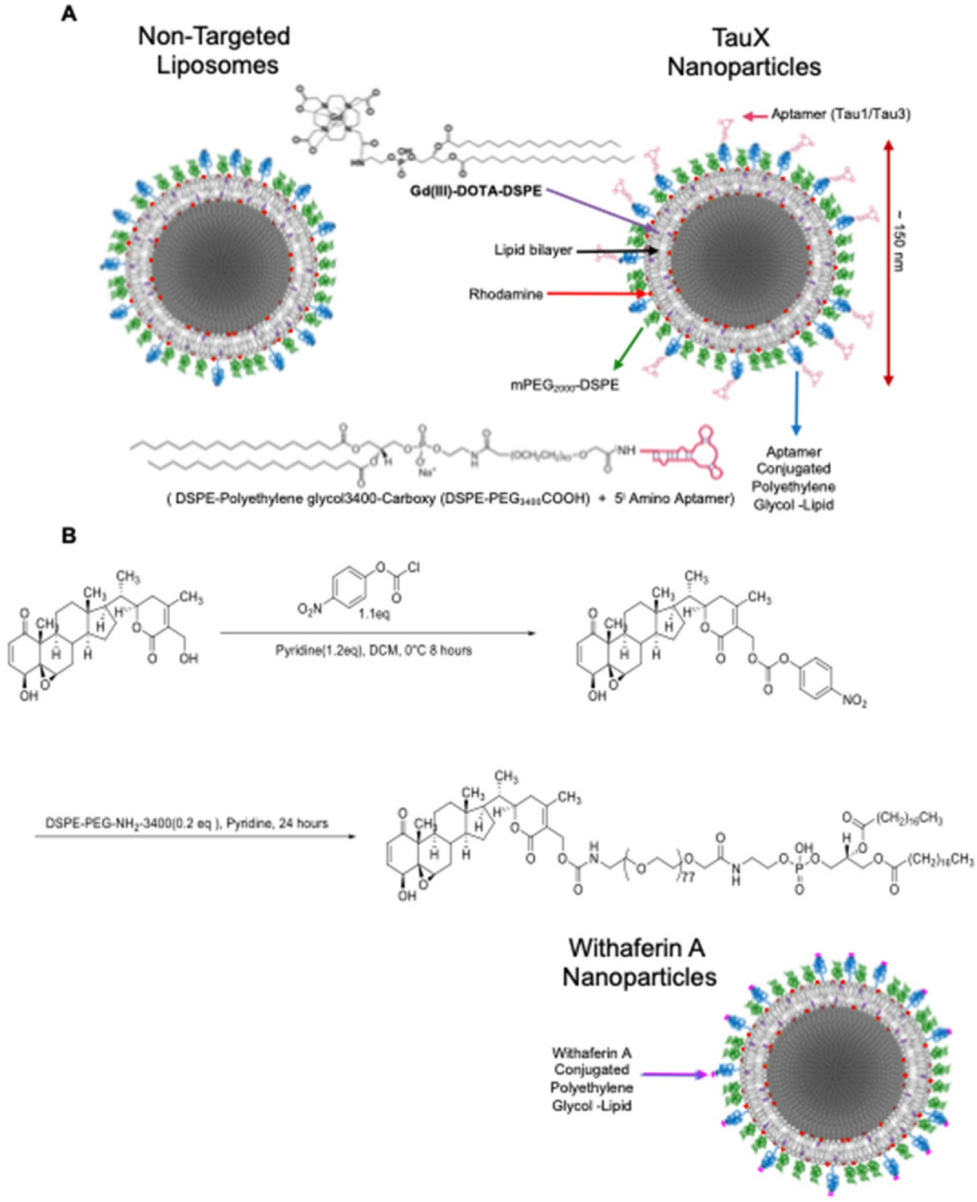
** Targeted liposomal-Gd nanoparticle contrast agent. (A)** The liposome bilayer incorporates DSPE-DOTA-Gd for MR contrast, lissamine rhodamine for fluorescence imaging, DSPE-mPEG2000 to enhance circulation half-life, DSPE-mPEG3400 for non-targeted (control/stealth) liposomes and DSPE-PEG3400-aptamer (Tau1/Tau3) for targeted TauX nanoparticles. **(B)** Synthesis of lipidized Withaferin A. Activation of the Withaferin A primary alcohol with 4-nitrophenyl chloroformate was performed to obtain 4-nitrophenyl Withaferin A carbonate that was reacted with heterobifunctional DSPE-PEG-NH2 to yield Withaferin A-DSPE-PEG3400 conjugate used in the preparation of Withaferin A nanoparticles (WNP).

For *in vivo* imaging of hyperphosphorylative state in the brains of live mice, two types of aptamer-targeted nanoparticles were developed as M-MRI contrast agents. One displayed the Tau1 aptamer (TauT1) and the other displayed the Tau3 aptamer (TauT3). PEGylated liposomal nanoparticles were synthesized using a lipid mixture that included lipid-tethered-DOTA-Gd for MR imaging and lissamine-rhodamine as a fluorescent tag 24,25,35. Particles had a hydrodynamic diameter of ~150 nm, ~86,000 Gd-chelates per liposomes and ~500 aptamers conjugated to the outer leaflet of each liposomal nanoparticle (Figure **5**).

### *In vivo* molecular MRI using TauX for detection of hyperphosphorylative cells

**Figure 6:**
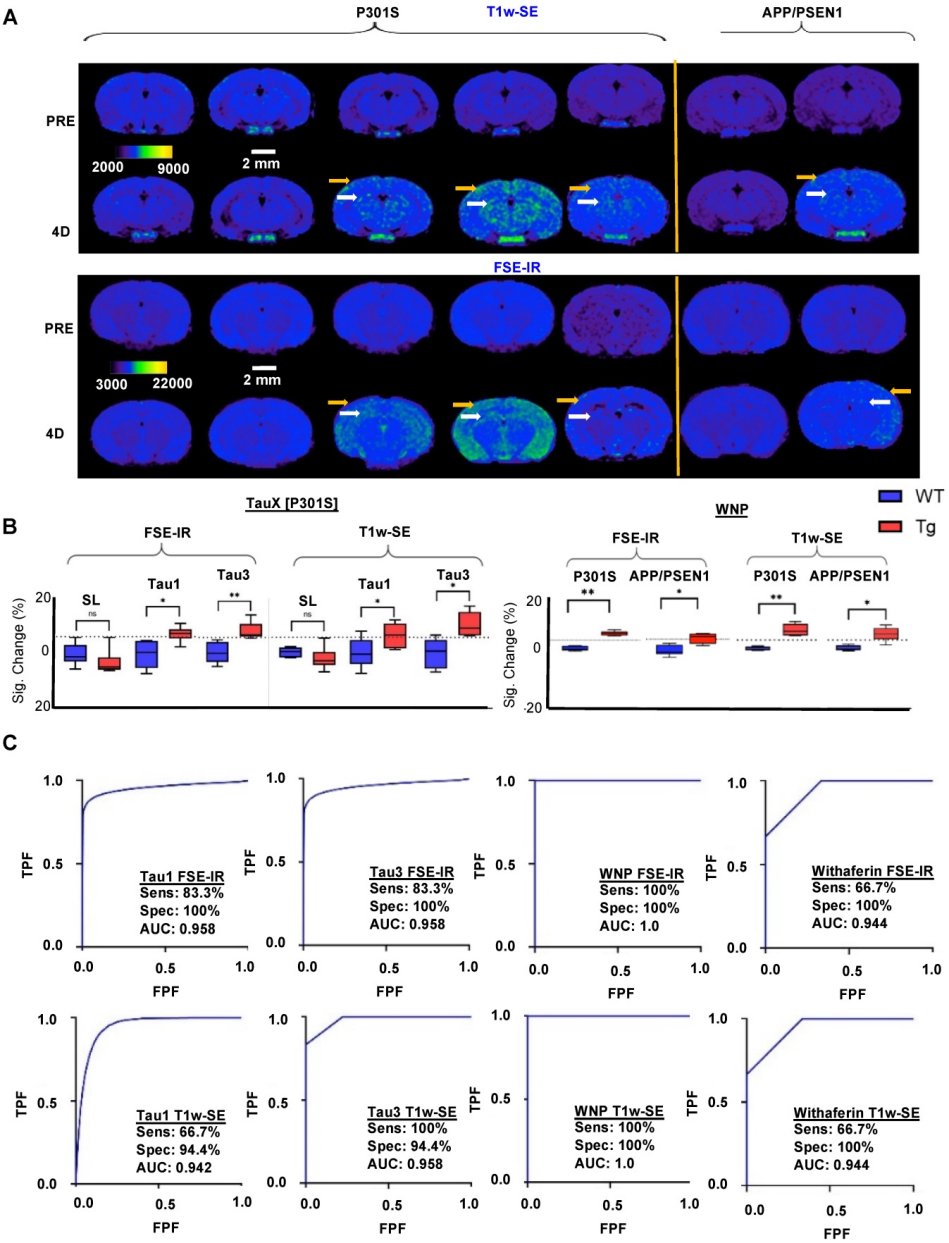
** Magnetic resonance imaging (MRI) and immunofluorescent staining demonstrate signal enhancement as TauX binds to hyperphosphorylated tau *in vivo*. (A)** Two-month-old mice were intravenously injected with TauX or WNPs (0.15 mmol Gd/kg). Pre- and post-contrast images for T1-weighted spin echo (T1w-SE) and fast spin echo inversion recovery (FSE-IR) demonstrate signal enhancement in delayed post-contrast scans of transgenic (TG) P301S mice treated with TauX or WNP, and APP/PSEN1 mice treated with WNP, relative to age-matched wild type (WT) controls. TG animals showed high enhancement in cortical (yellow arrow) and hippocampal regions (white arrow). TG animals showed no signal enhancement four days after injection of untargeted contrast (SL). Scale bar represents 2 mm. All animals are shown on the same color scale. **(B)** Box and whisker plots demonstrate signal enhancement in TG animals relative to WT counterparts and SL-treated TG animals for both T1w-SE and FSE-IR sequences (*p<0.05; **p<0.005). Dotted line indicates signal threshold for determining sensitivity (2 standard deviations above baseline noise, ~6%). **(C)** Receiver operating characteristic (ROC) curves plotting true positive fraction (TPF) against false positive fraction (FPF) demonstrate TauX and WNP accuracy in identifying early age TG animals. A fitted curve (blue) connects the observed operating points. Area under curve (AUC) is calculated using the fitted curve, and sensitivity (true positive rate) and specificity (true negative rate) for both formulations are listed. *SL = Stealth Liposomes with no targeting moiety.

Studies were performed in P301S transgenic and age-matched wild type mice at 2-3 months of age. At this age, transgenic animals do not show frank tau pathology, but practically all will develop tau pathology by 8 months of age (Supplementary Figure **S4**). Animals underwent baseline, pre-contrast MRI (Supplementary Figure **S5A,** Figure **6A**). Thereafter, animals were intravenously administered MRI contrast agent (TauT1, TauT3, or non-targeted control stealth liposomes) (Supplementary Figure **S5A**). Delayed post-contrast MRI was performed 4 days later (Supplementary Figure **S5A**, Figure **6A**); images were acquired using a T1-weighted spin-echo (T1w-SE) sequence and a T1-weighted fast spin echo inversion recovery (FSE-IR) sequence 25. Transgenic mice administered TauT1 and TauT3 demonstrated signal enhancement in the cortex and the hippocampus regions of the brain (Figure **6A, 6B**). Wild-type mice (WT) administered TauT1 or TauT3 targeted Gd-lips did not show signal enhancement in the brain. Similarly, transgenic mice administered non-targeted liposomal-Gd contrast agent did not show signal enhancement in cortex or hippocampus. These regions of interest were further analyzed quantitatively and signal-enhancement between the transgenic and wild-type mice were found to be statistically significant (p < 0.05). A baseline enhancement threshold of ~6% (=2X standard deviation of signal in baseline scans) was used as the classification threshold. Animals that showed signal enhancement above the threshold were identified as positives. Receiver operating characteristic (ROC) curves were generated with a six‐point ordinal scale to assess sensitivity and specificity for detecting the genotype, using TauT1 and TauT3 targeted nanoparticle contrast agents, and constructed over the entire tested group, including controls (Figure **6C**). The aptamer-targeted nanoparticle contrast agents, TauT1 and TauT3, showed overall AUC and accuracy of ∼0.95. TauT3 demonstrated higher sensitivity than TauT1.

Post-mortem brain analysis was performed in 2-3-month-old transgenic and age-matched non-transgenic siblings. Immunofluorescence analysis using the AT8 antibody revealed the presence of hyperphosphorylated tau species in transgenic mice but not in wild type mice (Supplementary Figure **S5B**). A 100% concordance was observed between AT8 positivity and animal genotype.

### Vimentin targeted Withaferin nanoparticles (WNP) for magnetic resonance imaging

Liposomal nanoparticles targeting Vimentin were also prepared using Withaferin A, a small molecule that binds the conserved cysteine 382 residue on tetrameric vimentin in a binding pocket that includes Gln 324 and Asp 331 36. We synthesized DSPE-PEG3400-Withaferin A substituting it for the carboxy PEG in the aptamer-targeted TauX formulation to yield Withaferin Gd liposomal nanoparticles (WNP) with ~600 Withaferin A molecules per liposome (Figure **5B**). Specific binding of WNP's to neuronally differentiated SH-SY5Y cells under hyperphosphorylative conditions is shown in Supplementary Figure **S6**. Cryo-EM analysis of WNP and TauX nanoparticles demonstrated typical liposomal morphology as evidenced by spherical shape and presence of unilamellar bilayer visible by transmission electron microscopy (Supplementary Figure **S7)**. Two-month-old P301S and APP/PSEN1 mice were injected with WNP and imaged using the same T1-weighted sequences as used with the TauX nanoparticles. No signal enhancement was observed in both WT mice models whereas the transgenic mice (P301S and APP/PSEN1) showed distinct signal enhancement in the cortex and hippocampus regions and were identified as positives (Figure **6A-B**). Group statistical analysis (Figure **6B-C**) revealed that the vimentin-targeted WNP contrast agents, showed overall AUC and accuracy of ∼1.00. The phosphorylation status of tau in transgenic mice was confirmed by immunofluorescence (Supplementary Figure **S8**).

## Discussion

The ATN research framework suggests the need for biomarkers to diagnose and classify AD. Under this framework, CSF based detection of Aβ, tau (total, and phosphorylated) have been reported but only at the prodromal stage of disease, in patients with mild cognitive impairment 37. Non-invasive neuroimaging tools, such as structural MRI to diagnose and monitor neurodegeneration 38 show a definitive correlation with cognitive decline, visualizing atrophic regions that depict neuronal injury in late-stage disease 39. However, a reliable combination of markers of early pre-symptomatic disease is yet to be identified.

While the role of Aβ and tau in the development of AD and the mechanism of transition from pre-symptomatic to symptomatic AD are yet unclear, the time scale of the transition is generally accepted to be as long as 10-20 years 14. Aβ deposits are considered the start of neurodegeneration but recent studies indicate that tau pathology 40 shows a stronger correlation with disease progression suggesting that the limitation of current tests is their inability to identify early-stage pathological tau 15. CSF presence of hyperphosphorylated tau species p-181 and p-217 is associated with Aβ deposition that precedes a positive tau PET 41 but only has a concordance of 50%-70% 42. Taken together, the roles of Aβ and tau deposition in disease progression and the role of Aβ in the spread of initial tau aggregates, strongly suggest that a biomarker of pathological tau at a pre-symptomatic stage of the disease is likely to advance detection by several years and constitutes the motivation for this work.

Initial tau aggregation is thought to be triggered by an imbalance in cellular homeostasis caused by dysregulated phosphorylation 43,44. Several kinases can phosphorylate tau at multiple locations; of the 80 sites theoretically identified, phosphorylation has been observed on at least 45 45-47. Combined with reduced phosphatase activities in AD, the altered kinase-phosphatase balance yields hyperphosphorylative conditions that cause abnormal hyperphosphorylation of tau 48. Disruption of the normal function of tau, modulating microtubule dynamics by lowering its binding capabilities 49 increases the level of cytosolic free tau leading to aggregation and fibrillization of tau that spreads throughout the connected brain, seeding pathology 50,51. We hypothesized that this initial process of hyperphosphorylation is associated with changes on the surface of hyperphosphorylative cells. We therefore sought to identify these surrogate markers of tau hyperphosphorylation that presage future tau pathology.

Using SH-SY5Y cells as a model of neuronal hyperphosphorylation, we used a reverse-phase protein array (RPPA) analysis to demonstrate elevated levels of surface molecules specific to the hyperphosphorylative state (Figure **1C**). Cell-SELEX capturing the differences between the surface of hyperphosphorylative cells and normal cells allowed the selection of phosphorothioate modified short DNA aptamers that bound with high affinity and specificity to hyperphosphorylative cells (Figure **2A**). We then used these aptamers as binding ligands and developed MR molecular imaging contrast agents that recognize the surface of cells in a hyperphosphorylative state. SH-SY5Y cells are not true neurons, they are a cell line originating in a neuroblastoma, a tumor of embryological neural crest origin, -derived cell line that has been used as a model system to study tau biology. However, they can be induced to differentiate to a neuronal phenotype (as in the current work). While primary neuronal culture or immortalized neuronal cells such as ReN-VM may offer alternative models of neurons to validate the aptamers *in vitro*, we showed that these aptamers have high affinity for hyperphosphorylative ReN-VM cells. We have functionally tested the aptamer hits from our SELEX screen in a transgenic mouse model of tau deposition, and validated their performance, supporting our position that the choice of cell model was adequate to identify suitable markers of tau hyperphosphorylation.

We have narrowed down the possible binding targets of the aptamers, and our data suggest that cell surface vimentin is a potential target. We have confirmed the specific presence of cell surface vimentin on the surface of SH-SY5Y cells in a hyperphosphorylative state, and on P301S mouse brain sections. Vimentin is an intermediate filament protein that undergoes constant assembly and/or remodeling and is usually associated with mesenchymal cells 52. The assembly state of filaments is linked to their phosphorylation state, phosphorylation promotes disassembly 53. Vimentin contains more than 35 phosphorylation sites targeted by multiple kinases and phosphatases allowing it to adjust IF dynamics dependent on its environment 54,55. Mechanical, chemical (toxins, hypoxia), and microbial stresses upregulate vimentin and its phosphorylation that allows cells to adjust their mechanical properties 56,57. The balance of different oligomeric forms influences dynamic cell processes including adhesion, migration, and invasion including stress-induced signaling 58. Vim IF's (~10 nm) distributed throughout the cell by association with microtubules (tubulin, 24 nm) regulating cell-migration, and microfilaments (actin, 7 nm) regulating cell-contractility, form the cytoskeletal network and provide mechanical support for the plasma membrane where it contacts other cells or the extracellular matrix 59. Interestingly, during the process of epithelial to mesenchymal transition wherein non-motile, polar epithelial cells transform to motile invasive non-polar mesenchymal cells 60, cells also undergo a cytoskeletal reorganization that includes changes in cell-membrane integrity, disassembly of junction proteins, increased stress-fiber formations, and altered cell-surface protein expression. Changes in the localization of proteins are a hallmark of this pathologic process. Our observation that vimentin is upregulated and translocated to the cell surface 61 in the early stages of tau hyperphosphorylation suggests a possible role for EMT-related processes at the start of a slow progression towards AD pathology.

PET is the leading modality for clinical molecular imaging, driven by its high contrast sensitivity; however, it suffers from poor spatial resolution on the order of 5-10 mm, high cost, limited access to radioactive tracers, and radiation exposure. Nanoparticle-enhanced MR imaging overcomes all these obstacles, but historically has not achieved high enough sensitivity. We have previously demonstrated liposomal nanoparticles exhibiting large numbers of Gd chelates in the external bilayer leaflet, with hyper-T1 relaxive properties resulting in contrast sensitivity that rival nuclear imaging 24,62, and took advantage of this platform in the current work, to create molecular MRI agents for the identification of very early-stage tau pathology. An often-quoted concern about the use of nanoparticles for brain imaging centers on whether these particles can penetrate the blood-brain barrier (BBB). The notion of the BBB arose primarily in the context of delivery of relatively large amounts of therapeutic molecules to brain tumors. For imaging however, relatively small amounts of contrast agent need to be delivered. AD imaging agents enter the brain via two distinct pathways either through the choroid plexus or permeable BBB. The choroid plexus is a highly vascularized tissue, it is distinct from the BBB in that the capillaries in the choroid plexus are highly fenestrated (up to 350 nm) and devoid of tight junctions 63-65, enabling non-selective passage of macromolecules and nanoparticles. Growing evidence from clinical studies also suggests that the choroid plexus may play an important role in pathogenesis of Alzheimer's disease as evidenced by morphological and functional changes 66,67. We have previously demonstrated using ultra-high resolution computed tomography (CT) imaging and a non-targeted liposomal-iodine nanoparticle contrast agent that nanoparticles entered the brain predominantly via the choroid plexus 68. Although, an impaired BBB may serve as a route for brain entry in AD, a leaky choroid plexus constitutes the major route for nanoparticle entry in the brain of healthy and AD mice, consistent with the known biology of choroid plexus and its role in brain transport. We performed a similar nanoparticle contrast-enhanced CT study in young (2-3 months old) wild type and transgenic P301S mice and confirmed that choroid plexus is the dominant route, independent of age and genotype, for nanoparticle leakage in the brain (Supplementary Figure **S9**).Taking advantage of this route, we have previously demonstrated a liposomal MRI agent targeting amyloid plaque that successfully enters the brain following intravenous injection and binds plaques enabling imaging of amyloid pathology by T1-weighted MRI analogous to the current work using multiple models of AD as well as varying mouse age (6 - 18 months) 25,69,70. The choroid plexus route is the most likely route for the M-MRI particles of the current work to enter the brain and bind their targets.

In P301S mice, the earliest reported histopathological studies are at an age of 2.5 months 71,72, and report no tau pathology. “Tau seeding”, the cell-cell transfer of pathogenic tau aggregates has been reported using brain homogenates at 1.5 month of age 73 suggesting that conditions supportive of pathological tau exist at this early age. We chose P301S mice at 2 months of age for our studies when tau seeding could be taking place but frank tau pathology is absent. The mice were injected with TauX nanoparticles targeted either by the Tau1 aptamer or the Tau3 aptamer. When imaged by T1-weighted MRI sequences, designed to optimize signal from the Gd-chelate induced T1 relaxation caused by the liposomal-Gd nanoparticles, signal enhancement was observed in the cortex and hippocampus regions of the brain. T1w-SE images demonstrated the greatest changes in hippocampal structures while FSE-IR sequences, which have thicker imaging slices were more sensitive to cortical signal increase. Hyperphosphorylative conditions were confirmed by post-mortem IF staining with the AT8 antibody that recognizes the S202 and T305 pTau species. Signal enhancement was not observed in non-transgenic mice, or in transgenic mice injected with untargeted nanoparticles, supporting the specificity of Tau1-or Tau3-bearing nanoparticle binding to target. We provide validation of our vimentin binding TauX nanoparticles by the use of a small molecule, Withaferin A known to bind vimentin at its highly conserved cysteine residue in coiled-coil 2B domain 36,74. Withaferin targeted nanoparticles when injected intravenously exhibited binding in the same brain regions as TauX and maintained specificity and sensitivity to the phosphorylative state, with signal enhancement observed only in transgenic mice and not in WT mice. Additionally, we also show similar results in another mouse model, APP/PSEN1, used to study amyloid plaque formation and has been shown to contain phosphorylated tau neuritic processes but no mature tau pathology 75. Notably the amyloid plaques in APP/PSEN1 mice are also immunoreactive for hyperphosphorylated tau 76. Molecular events that represent hyperphosphorylation are reported from an early age 77-79. Evidence of hyperphosphorylation in APP/PSEN1 mice at 2 months of age is shown by staining phosphorylated tau species (Supplementary Figure **S10**). Taken together, this *in vivo* data further supports the use of such particles as detectors of hyperphosphorylation that leads to the initiation of tau pathology in AD. We also confirmed higher expression of CSV in transgenic P301S mice at 2 months of age by immunofluorescence (Supplementary Figure **S11**).

While positron emission tomography (PET) is the mainstay of molecular imaging, and exhibits remarkable sensitivity, it does have significant limitations 62,80. Access to PET imaging is limited, even in the relatively well-served US, and is skewed towards high density urban centers and academic medical facilities. PET costs are very high due to the rapid decay of the isotopes and resultant need for same-day radiosynthesis. Longer half-life isotopes cause higher radiation exposure. This tradeoff between half-life and radiation exposure greatly limits the reach of PET to a wider patient population. Current PET tau tracers recognize the tau β-sheets in the PHF and NFT present in tauopathies 81. This conformation is not unique to tau and the *in vivo* specificity is circumspect limiting its interpretation. Off-target binding of Flortaucipir, an approved tau PET agent, has been reported since it binds the MAO-B enzyme in the brain 82,83. Further, the vast majority of pathological tau is intracellular, posing a significant barrier to PET tracers that must navigate to the site of tau pathology, bind the target, and have all unbound tracer molecules cleared from the brain before the radioactive signal decays. Our choice of MRI as the detection modality is based on T1 hyper-relaxive properties of nanoparticles with surface conjugated Gd chelates, bringing detection sensitivity to the same range as nuclear imaging, and the MRI agent does not suffer from the rapid signal decay of PET agents, allowing plenty of time for unbound tracer to clear from the brain before imaging. One potential limitation of molecular MRI, however, is the relatively low apparent signal for tracking imaging probes. To overcome this challenge, we utilized robust methods for characterizing signal change between Tg and WT mice and employed both a T1w-SE sequence which had better out-of-plane resolution (1.2 mm slice thickness) and an FSE-IR sequence with thicker slices (2.4 mm slice thickness). The use of both sequences allows for greater sensitivity in the cortex (FSE-IR) and investigation of additional brain structures (T1w-SE). The difference in slice thickness, however, leads to different signal enhancement profiles which is why data for both sequences are listed individually. Our choice of a cell surface surrogate marker of tau hyperphosphorylation avoids the need to bind an intracellular target. Finally, MRI imaging is already included in AD management and can be adjusted with agents such as TauX nanoparticles to constitute a highly sensitive and specific test for future tau pathology.

In summary, our work shows that the hyperphosphorylative conditions coinciding with the initiation of a decades long process culminating in AD result in markers manifested on the cell surface. By imaging their presence, our approach detects the presence of hyperphosphorylative cells indicative of a presymptomatic stage of AD in mouse models. If the results translate to humans, enabling such early detection could potentially advance the diagnosis of AD by several years.

## Materials and Methods

**Cell-lines -** SH-SY5Y cells (ATCC, Manassas, VA, #CRL-2266™) were obtained from Dr. Jason Shohet's lab at the Texas Children's Hospital, Houston, immortalized human hepatocytes (THLE-3) were purchased from American Type Culture Collection (ATCC, Manassas, VA, # CRL-11233^TM^); both were cultured according to the ATCC instructions. ReN cell™ VM (#SCC008) cultured as per instruction using neural stem cell maintenance medium (#SCM005) and growth factors EGF (GF001) and bFGF(#GF005) all from Millipore Sigma, Burlington, MA.

**Differentiation -** SH-SY5Y cells were exposed to 30 µM *all-trans*-Retinoic acid (Sigma-Aldrich, St.Louis, MO, # R2625) in serum free cell medium for 10 days with medium change every alternate day. ReNcell VM were differentiated by the removal of growth factors from its culture medium for 10 days.

**Hyperphosphorylation -** was induced in SH-SY5Y cells by addition of 30 nM okadaic acid (Sigma Aldrich, St.Louis, MO, # 459620) in growth medium with 30 µM RA for 24 h. ReNcell VM were hyperphosphorylated using 100 nM Quinolinic Acid (Sigma Aldrich, St. Louis, MO, #P63204) in culture media for 24 h.

**Synthesis of primers and TA DNA library -** All primers and Cy5, and amine labelled selected aptamers were purchased from Integrated DNA Technologies (IDT, Coralville, IA). The ssDNA library used in Cell-SELEX contained a central randomized sequence of 30 nucleotides flanked by PCR primer regions to enable the PCR amplification of the sequence (5'-CGCTCGATAGATCGAGCTTCG-(N)_30_-GTCGATCACGCTCTAGAGCACTG-3'). The chemically synthesized DNA library was converted to a phosphorothioate modified library by PCR amplification using, dATP (αS), resulting in the DNA sequences where the 3' phosphate of each residue is substituted with monothiophosphate groups, as described previously in detail 22,84. The reverse primer was labeled with biotin to separate the sense strand from the antisense strand by streptavidin-coated sepharose beads (PureBiotech, Middlesex, NJ, # MSTR0510) for the next selection round. The concentration of the TA library was determined with a NanoDrop™ 2000 by measuring the UV absorbance at 260 nm.

**Cell-SELEX -** The procedures of Cell-SELEX were modified from protocols described previously 23,85.The initial ssDNA library of 150 pmole was dissolved in binding buffer with a total volume of 350 µl. It was denatured by heating at 95°C for 5 min, then annealed by rapid cooling on ice for 10 min. The treated SH-SY5Y cells at approximately 90% confluence in a 100-mm culture plate were washed twice with washing buffer and followed by incubating with the ssDNA library of 150 pmole for 2 h at 4 °C. Following the incubation, for positive selections, the supernatant was removed, and cells were washed three times with washing buffer to remove any unbound sequences. Then cells were scraped off and transferred to nuclease-free water, following another three times nuclease-free water washes. Cells in nuclease-free water was centrifuged at 300×g for 5 min. QIAamp DNA Mini and Blood Mini kit (Qiagen, Germantown, MD, # 51104) was introduced to elute cell membrane fraction. The cell membrane fraction was PCR-amplified to monitor the presence of cell binding efficacy at each cycle. For negative selections, the supernatant was simply pipetted out of the flask and processed for the next cycle of selection. The desired compartment was amplified by PCR and used to prepare the TA for the next round of selection. Two different negative selections were involved. One was differentiated treatment only SH-SY5Y cells at cycles #12 and #13. Another was hepatocyte THLE-3 cells at cycles #20 and #21. A total of 26 cycles of Cell-SELEX were conducted, including two different types of negative selections mentioned above.

**Next-Generation Sequencing (NGS) -** At the studied cycles, the membrane fractions were isolated and the recovered TA sequences were amplified by PCR. Equimolar quantities of the recovered TA sequences over the range were pooled together and sequenced by Next-Gen DNA sequencing using Ion318 chip (ThermoFisher, Waltham, MA). A four base sequence was introduced during PCR amplification to serve as unique “barcode” to distinguish between the studied cycles. Sequencing results were analyzed by the Aptaligner 30 that uses a Markov model approach to find the optimal alignment of the sequences.

**Aptamer binding studies** - were conducted with undifferentiated, differentiated and hyperphosphorylated SH- SY5Y and ReN cell VM grown in 96-wells seeded at 10000 per well. The apparent dissociation constants (Kds) were measured by the equation Y=Bmax X/ (Kd+X), with GraphPad Prism 9, San Diego, CA, with a saturation binding experiment; cells were incubated with varying concentrations of Cy5-labeled aptamer in a 100 μl volume of binding buffer containing cells, incubated for 30 min, washed twice and resuspended in 100 μl buffer and analyzed by Molecular Devices (San Jose, CA) microplate reader equipped with the appropriate excitation and emission filters. All data points were collected in triplicate.

**Target Identification -** The protein targets of Tau-1, Tau-3, Tau-4 and Tau-5 were identified by affinity-pull down using the selected aptamers as the capturing reagent followed by mass-spectroscopy. A scrambled DNA sequence, R2, was used as a control. The hyperphosphorylated SH-SY5Y cells at 90-95% confluence were washed with cold PBS buffer and incubated with biotinylated selected aptamers with 25mmol/l each at 4 °C in PBS, respectively. After 2 h of gentle agitation, SH-SY5Y cells were cross-linked with 1% formaldehyde for 10 minutes at room temperature. The formaldehyde cross-linking was quenched with glycine. Cells were scraped from the plate, washed and lysed with lysing buffer (Thermofisher Scientific, # 87787) and treated with protease inhibitor mixture. The lysates were freeze-thawed for 30 minutes on ice and cleared by centrifuging at 10,000 ×g for 2 min at 4 °C. To pull down the cross-linked proteins, equal amounts of cell lysate were incubated with prewashed streptavidin magnetic beads for 1 h at room temperature with continuous rotation. Protein digestions were performed on the beads to isolate targeted proteins and processed for mass spectrometric analysis. Each sample was analyzed in triplicates. The raw data files were processed to generate a Mascot Generic Format with Mascot Distiller and searched against the SwissProt_2012_01 (Human) database using the licensed Mascot search engine v2.3.02 (Matrix Science, Boston, MA) run on an in-house server.

**Immunocytochemistry -** An eight-well glass plate was coated with a solution of 100 µg/ml Collagen Type I (Thermofisher Scientific, Waltham, MA, # A1064401) dissolved in 0.01N HCl and air dried, then PBS washed and air dried prior to seeding 20,000 SH-SY5Y cells per well. Cells were treated with 50 nM aptamers in reduced serum medium at 37 ⁰C for 2 hrs, followed by fixation under incubation for 15 min in 4% formaldehyde in PBS at room temperature. Non-specific binding was blocked with blocking buffer (G-Biosciences, St. Louis, MO, # 786195) for 1 h and overnight incubation at 4 °C with the rabbit pTau primary antibody (1:100) (Santa Cruz Biotechnology, #: sc-101815) was followed by washing with PBS, and 1h incubation with goat anti-rabbit IgG secondary antibody, Alexa Fluor 488 (Invitrogen, Carlsbad, CA, # A-11008) for 1 h at room temperature. Cytoskeletal actin filaments were stained with Alexa Fluor 594 Phalloidin (Invitrogen, # A12381). The cells were covered with VECTASHIELD hardset mounting medium with DAPI (Vector Laboratories, Burlingame, CA, # H-1500) for 5 min at room temperature. Images were visualized with an Olympus Fluoview FV1000 confocal microscope.

**TauX nanoparticle synthesis -** L-α-phosphatidylcholine, hydrogenated (Hydro Soy PC; HSPC) and Cholesterol were purchased from Lipoid Inc., Newark NJ, USA. 1,2-distearoyl-sn-glycero-3-phosphoethanolamine-N- [methoxy(polyethylene glycol)-2000] (DSPE-mPEG2000) was purchased from Corden Pharma, Liestahl, Switzerland. DSPE-PEG3400-COOH and Gd-DOTA-DSPE were synthesized *in house*, lis-rhodamine-DHPE from ThermoFisher Scientific. HSPC, Cholesterol, DSPE-PEG3400-COOH, DSPE-mPEG2000, Gd-DOTA-DSPE, lis-rhodamine-DHPE at molar proportions 31.4:40:0.5:3:25:0.1 were dissolved in ethanol. For the non-targeted control stealth liposomes, carboxy terminated PEG was not included in the lipid mixture. The ethanolic solution of lipids was hydrated with 150mM saline solution at 65 °C for 30 minutes, allowing multilamellar liposomes to form. The mixture was then extruded in a 10ml Lipex extruder (Northern Lipids Inc., Burnaby, Canada) using a 400 nm polycarbonate track-etch polycarbonate filter (3 passes) followed by a 200 nm (3 passes) and finally through 100 nm filters. The suspension was then diafiltered using a MicroKros cross-flow diafiltration cartridge (*500 kDa* cutoff) from Repligen (Rancho Dominguez, CA) exchanging the external buffer with phosphate buffered saline (PBS, pH 7.2) for 15 volume exchanges. To form the aptamer conjugated liposomes, liposomes with lipid-PEG-COOH were reacted with amine terminated aptamers using carbodiimide chemistry. The carboxyl groups on the liposomes were activated with 5 mM EDC and 10 mM sulfo-NHS at pH~6 for 5-10 min. The activated liposomes were then immediately reacted with the amine terminated aptamers and the pH was raised to ~7.3 - 7.6 by titrating µl amounts of 5 N NaOH. The final concentration of aptamers used in reaction is ~140 µM. The reaction was mixed at room temperature for 1 hfollowed by stirring at 4 °C overnight. The liposomes were then dialyzed against PBS to remove unconjugated aptamers using a 300 kDa dialysis membrane. The dialysate (external phase) was concentrated using 10 kD centrifugal separator and washed with PBS to remove residual EDC/s-NHS. The concentrated dialysate was analyzed by NanoDrop Spectrophotometer (ThermoFisher Sci., Waltham, MA, USA) to determine unconjugated aptamer fraction, and estimate aptamer density per nanoparticle in TauX formulations. Inductively coupled plasma atomic emission spectroscopy (ICP-AES) was used to measure Gd and phosphorus concentrations of TauX formulations. The hydrodynamic diameter of liposomal nanoparticles in TauX formulations was determined using a dynamic light scattering instrument.

**Withaferin A nanoparticle (WNP) synthesis -** The allylic alcohol of Withaferin A was selectively activated by exposure to 4-nitrophenyl chloroformate (1.1 eq) at 0 ⁰C for 8 h to give intermediate compound B in excellent yield. This was then reacted with DSPE-PEG-NH2-3400 at room temperature for 24 h. The crude product was dialyzed against water for 2 days, and then freeze dried to yield DSPE-PEG-3400-Withaferin A. Structures of the intermediate and final product were confirmed by NMR and MALDI. Liposomal nanoparticles containing Withaferin A on its surface were generated by using the TauX composition substituting the carboxy PEG with the synthesized DSPE-PEG3400-Withaferin A to yield Withaferin nanoparticles (WNP).

**Mice -** All the procedures were performed with approval from Institutional Animal Care and Use Committee (IACUC) of Baylor College of Medicine. Mice were kept under a 12 h light/dark cycle, with food and water available ad libitum. PS19 mice from Jackson Laboratories (Bar Harbor, ME) B6; C3-Tg (Prnp-MAPT*P301S) PS19Vle/J Stock No: 008169 were used and experiments were conducted at the 2 months of age. The transgenic (TG) mice develop neurofibrillary tangles by 5 months of age 72. Age-matched non-transgenic wild type (WT) mice were used as controls. APP/PS1 mice from Jackson Laboratories B6.Cg-Tg(APPswe,PSEN1dE9)85Dbo/Mmjax MMRRC Stock No: 34832-JAX at 2 months of age were also used for experiments with WNP. These mice generate amyloid plaques by 6weeks in cortex and 2-4 months in hippocampus without any reported mature tau tangles but presence of hyperphosphorylated tau neuritic processes has been observed around plaques 75.

**Magnetic Resonance Imaging (MRI) -** MRI was performed on a 1T permanent magnet scanner (M7, Aspect Imaging, Shoham, Israel). Mice underwent pre-contrast baseline scans. Thereafter, mice were intravenously administered one of three nanoparticle MR contrast agents (TauT1, TauT3 or non-targeted control liposomes) via tail vein at a dose of 0.15 mmol Gd/kg of body weight. Delayed post-contrast MRI was performed 4 days after contrast agent injections. Pre-contrast and delayed post-contrast MR images were acquired using a T1-weighted spin echo (T1w-SE) sequence and a fast spin echo inversion recovery (FSE-IR) sequence with the following parameters: *SE parameters*: TR = 600 ms, TE = 11.5 ms, slice thickness = 1.2 mm, matrix = 192 × 192, FOV = 30 mm, slices = 16, NEX = 4; *FSE-IR parameters*: TR = 13500 ms, TE = 80 ms, TI = 2000 ms, slice thickness = 2.4 mm, matrix = 192 × 192, FOV = 30 mm, slices = 6, NEX = 6. Coil calibration, RF calibration, and shimming were performed at the beginning of study for each subject. The pre-contrast scans provide a baseline for calculation of signal enhancement from resulting post-contrast scans 25. Two-standard deviations above the mean variation within WT control animals were used as the cutoff signal intensity for identifying tau positive animals. Six transgenic (TG) mice and six wild type mice (WT) were used for testing of each nanoparticle contrast agent formulation. Receiver operating characteristic (ROC) curves were generated on a six-point ordinal scale by plotting the true positive fraction (TPF) against the false positive fraction (FPF) based on imaging-based identification of Tau-positive animals using the cutoff signal intensity and then comparing against histological confirmation of Tau pathology as a gold standard. A fitted curve was then generated against the empirical points plotted on the graphs. Qualitative and quantitative analysis of MRI images was performed in OsiriX (version 5.8.5, 64-bit, Pixmeo SARL, Geneva, Switzerland) and MATLAB (version 2015a, MathWorks, Natick, MA).

**Computed Tomography (CT) Imaging:** Inorder to evaluate brain uptake of nanoparticles in young mice, a subset of 2 months old mice (n=3/genotype) underwent contrast-enhanced computed tomography (CECT) imaging using a liposomal-iodine (Lip-I) nanoparticle CT contrast agent as per methods described in our previous work 68. Mice underwent baseline non-contrast CT scans. Lip-I contrast agent was intravenously injected via tail vein at a dose of 2 mg I/g body weight and delayed post-contrast CT scans were acquired 7-8 days later. CT images were calibrated for Hounsfield unit (HU). The amount of nanoparticle leak in brain was estimated from CT images using measured leak volume, nanoparticle-associated signal enhancement and known normalization factor (~46 HU/mgI/mL) as per methods described in our previous work 68. Nanoparticle leak was reported in mg iodixanol (active ingredient in Lip-I contrast agent) expressed as mean and standard deviation.

**Immunofluorescence -** After the final MRI scan, the mice were euthanized and perfused extensively with 0.9% saline followed by 4% paraformaldehyde for 15 min. The brains were then immersion-fixed in 4% formaldehyde for 48 h at 4 ⁰C, transferred to 30% sucrose for cryoprotection and embedded in OCT. Phenotypic confirmation for the presence of phosphorylated tau and vimentin was done on 25 µm thick brain sections. Antigen retrieval in pH=8.5 citrate buffer was executed in a 1200W GE microwave for 15 min. After 15 min of cooling 25 µL of 1:50 dilution of primary p-tau antibody namely either AT8, or AT100 that recognizes p-tau Ser202/Thr205 or Thr212/Ser214 species were incubated in a tray (RPI, Mt. Prospect, IL #248270) designed for microwave enhanced immunostaining procedures for 3 min at power level 3. After a 2 min cooling, sections were washed with PBS and incubated for 3 min with a 1:100 dilution of appropriate secondary antibody. DAPI staining proceeded after 2 min of cooling and a PBS washing. ProGold Antifade (Invitrogen, Carlsbad, CA, # P36030) was used to mount slides which were visualized on Olympus Fluoview LV100. Scanning of whole sections was also conducted using a Biotek Cytation 5 slide scanning microscope. List of Antibodies - AT8 (Thermo Fisher Scientific, Waltham, MA, #MN1020), Vimentin SP20 (Thermo Fisher Scientific, Waltham, MA, #MA516409), Vimentin D21H3 (Cell Signaling Technology, Beverly, MA, #5741T,), Cell-surface vimentin (Abnova, Taipei City, Taiwan, #H00007431-M08J).

## Supplementary Material

Supplementary figures.

## References

[1] Goedert M, Wischik CM, Crowther RA, Walker JE, Klug A (1988). Cloning and sequencing of the cDNA encoding a core protein of the paired helical filament of Alzheimer disease: identification as the microtubule-associated protein tau. Proc Natl Acad Sci U S A.

[2] Goedert M, Spillantini MG, Jakes R, Rutherford D, Crowther RA (1989). Multiple isoforms of human microtubule-associated protein tau: sequences and localization in neurofibrillary tangles of Alzheimer's disease. Neuron.

[3] Iqbal K, Liu F, Gong C (2016). Tau and neurodegenerative disease: the story so far. Nat Rev Neurol.

[4] Guo T, Noble W, Hanger D (2017). Roles of tau protein in health and disease. Acta Neuropathol.

[5] Wang Y, Mandelkow E (2016). Tau in physiology and pathology. Nat Rev Neurosci.

[6] Chong FP, Ng KY, Koh RY, Chye SM (2018). Tau Proteins and Tauopathies in Alzheimer's Disease. Cell Mol Neurobiol.

[7] Karikari TK, Nagel DA, Grainger A (2019). Distinct conformations, aggregation and cellular internalization of different tau strains. Front Cell Neurosci.

[8] d'Errico P, Meyer-Luehmann M (2020). Mechanisms of Pathogenic Tau and Aβ Protein Spreading in Alzheimer's Disease. Front Aging Neurosci.

[9] Brunello CA, Merezhko M, Uronen RL, Huttunen HJ (2020). Mechanisms of secretion and spreading of pathological tau protein. Cell Mol Life Sci.

[10] Vogel JW, Iturria-Medina Y, Strandberg OT (2020). Spread of pathological tau proteins through communicating neurons in human Alzheimer's disease. Nat Commun.

[11] Gibbons GS, Lee VMY, Trojanowski JQ (2019). Mechanisms of Cell-to-Cell Transmission of Pathological Tau: A Review. JAMA Neurol.

[12] Jack Jr CR, Bennett DA, Blennow K (2018). NIA-AA research framework: toward a biological definition of Alzheimer's disease. Alzheimer's Dement.

[13] Jack CR, Bennett DA, Blennow K (2016). A/T/N: an unbiased descriptive classification scheme for Alzheimer disease biomarkers. Neurology.

[14] Selkoe DJ, Hardy J (2016). The amyloid hypothesis of Alzheimer's disease at 25 years. EMBO Mol Med.

[15] Mattsson-Carlgren N, Andersson E, Janelidze S (2020). Aβ deposition is associated with increases in soluble and phosphorylated tau that precede a positive Tau PET in Alzheimer's disease. Sci Adv.

[16] Leuzy A, Cicognola C, Chiotis K (2019). Longitudinal tau and metabolic PET imaging in relation to novel CSF tau measures in Alzheimer's disease. Eur J Nucl Med Mol Imaging.

[17] Wang J-Z, Xia Y-Y, Grundke-Iqbal I, Iqbal K (2013). Abnormal hyperphosphorylation of tau: sites, regulation, and molecular mechanism of neurofibrillary degeneration. J Alzheimer's Dis.

[18] Grundke-Iqbal I, Iqbal K, Tung Y-C, Quinlan M, Wisniewski HM, Binder LI (1986). Abnormal phosphorylation of the microtubule-associated protein tau (tau) in Alzheimer cytoskeletal pathology. Proc Natl Acad Sci.

[19] Delobel P, Lavenir I, Fraser G (2008). Analysis of tau phosphorylation and truncation in a mouse model of human tauopathy. Am J Pathol.

[20] Lund ET, McKenna R, Evans DB, Sharma SK, Mathews WR (2001). Characterization of the *in vitro* phosphorylation of human tau by tau protein kinase II (cdk5/p20) using mass spectrometry. J Neurochem.

[21] Liu F, Grundke-Iqbal I, Iqbal K, Gong C (2005). Contributions of protein phosphatases PP1, PP2A, PP2B and PP5 to the regulation of tau phosphorylation. Eur J Neurosci.

[22] Mu Q, Annapragada A, Srivastava M (2016). Conjugate-SELEX: A High-throughput Screening of Thioaptamer-liposomal Nanoparticle Conjugates for Targeted Intracellular Delivery of Anticancer Drugs. Mol Ther - Nucleic Acids.

[23] Parekh P, Tang Z, Turner PC, Moyer RW, Tan W (2010). Aptamers Recognizing Glycosylated Hemagglutinin Expressed on the Surface of Vaccinia Virus-Infected Cells. Anal Chem.

[24] Ghaghada KB, Ravoori M, Sabapathy D, Bankson J, Kundra V, Annapragada A (2009). New Dual Mode Gadolinium Nanoparticle Contrast Agent for Magnetic Resonance Imaging. PLoS One.

[25] Badachhape AA, Working PK, Srivastava M (2020). Pre-clinical dose-ranging efficacy, pharmacokinetics, tissue biodistribution, and toxicity of a targeted contrast agent for MRI of amyloid deposition in Alzheimer's disease. Sci Rep.

[26] Kamat PK, Rai S, Swarnkar S, Shukla R, Nath C (2014). Molecular and Cellular Mechanism of Okadaic Acid (OKA)-Induced Neurotoxicity: A Novel Tool for Alzheimer's Disease Therapeutic Application. Mol Neurobiol.

[27] Gulaj E, Pawlak K, Bien B, Pawlak D (2010). Kynurenine and its metabolites in Alzheimer's disease patients. Adv Med Sci.

[28] UniProt (2021). the universal protein knowledgebase in 2021. Nucleic Acids Res.

[29] Rothberg JM, Hinz W, Rearick TM (2011). An integrated semiconductor device enabling non-optical genome sequencing. Nature.

[30] Lu E, Elizondo-Riojas M-A, Chang JT, Volk DE (2014). Aptaligner: automated software for aligning pseudorandom DNA X-aptamers from next-generation sequencing data. Biochemistry.

[31] Zuker M (2003). Mfold web server for nucleic acid folding and hybridization prediction. Nucleic Acids Res.

[32] Katoh K, Rozewicki J, Yamada KD (2019). MAFFT online service: multiple sequence alignment, interactive sequence choice and visualization. Brief Bioinform.

[33] Sievers F, Wilm A, Dineen D (2011). Fast, scalable generation of high-quality protein multiple sequence alignments using Clustal Omega. Mol Syst Biol.

[34] Donato R, Miljan EA, Hines SJ (2007). Differential development of neuronal physiological responsiveness in two human neural stem cell lines. BMC Neurosci.

[35] Ghaghada K, Hawley C, Kawaji K, Annapragada A, Mukundan Jr S (2008). T1 relaxivity of core-encapsulated gadolinium liposomal contrast agents—effect of liposome size and internal gadolinium concentration. Acad Radiol.

[36] Bargagna-Mohan P, Hamza A, Kim Y (2007). The tumor inhibitor and antiangiogenic agent withaferin A targets the intermediate filament protein vimentin. Chem Biol.

[37] Mattsson N, Lönneborg A, Boccardi M, Blennow K, Hansson O (2017). Clinical validity of cerebrospinal fluid Aβ42, tau, and phospho-tau as biomarkers for Alzheimer's disease in the context of a structured 5-phase development framework. Neurobiol Aging.

[38] Coch RA, Leube RE (2016). Intermediate filaments and polarization in the intestinal epithelium. Cells.

[39] Cuingnet R, Gerardin E, Tessieras J (2011). Automatic classification of patients with Alzheimer's disease from structural MRI: a comparison of ten methods using the ADNI database. Neuroimage.

[40] Johansson M, Stomrud E, Insel PS (2021). Mild behavioral impairment and its relation to tau pathology in preclinical Alzheimer's disease. Transl Psychiatry.

[41] Suárez-Calvet M, Karikari TK, Ashton NJ (2020). Novel tau biomarkers phosphorylated at T181, T217 or T231 rise in the initial stages of the preclinical Alzheimer's continuum when only subtle changes in Aβ pathology are detected. EMBO Mol Med.

[42] Mattsson N, Schöll M, Strandberg O (2017). 18 F-AV-1451 and CSF T-tau and P-tau as biomarkers in Alzheimer's disease. EMBO Mol Med.

[43] Chung S-H (2009). Aberrant phosphorylation in the pathogenesis of Alzheimer's disease. BMB Rep.

[44] Oliveira J, Costa M, de Almeida MSC, da Cruz e Silva OAB, Henriques AG (2017). Protein phosphorylation is a key mechanism in Alzheimer's disease. J Alzheimer's Dis.

[45] Augustinack JC, Schneider A, Mandelkow E-M, Hyman BT (2002). Specific tau phosphorylation sites correlate with severity of neuronal cytopathology in Alzheimer's disease. Acta Neuropathol.

[46] Wang Y, Mandelkow E (2016). Tau in physiology and pathology. Nat Rev Neurosci.

[47] Wang J, Grundke-Iqbal I, Iqbal K (2007). Kinases and phosphatases and tau sites involved in Alzheimer neurofibrillary degeneration. Eur J Neurosci.

[48] Sontag J-M, Sontag E (2014). Protein phosphatase 2A dysfunction in Alzheimer's disease. Front Mol Neurosci.

[49] Sontag JM, Nunbhakdi-Craig V, White CL, Halpain S, Sontag E (2012). The protein phosphatase PP2A/Bα binds to the microtubule-associated proteins Tau and MAP2 at a motif also recognized by the kinase Fyn: Implications for tauopathies. J Biol Chem.

[50] Franzmeier N, Neitzel J, Rubinski A (2020). Functional brain architecture is associated with the rate of tau accumulation in Alzheimer's disease. Nat Commun.

[51] DeVos SL, Corjuc BT, Oakley DH (2018). Synaptic tau seeding precedes tau pathology in human Alzheimer's disease brain. Front Neurosci.

[52] Eriksson JE, Dechat T, Grin B (2009). Introducing intermediate filaments: from discovery to disease. J Clin Invest. 2009/07/01.

[53] Sihag RK, Inagaki M, Yamaguchi T, Shea TB, Pant HC (2007). Role of phosphorylation on the structural dynamics and function of types III and IV intermediate filaments. Exp Cell Res.

[54] Snider NT, Omary MB (2014). Post-translational modifications of intermediate filament proteins: mechanisms and functions. Nat Rev Mol Cell Biol.

[55] Battaglia RA, Delic S, Herrmann H, Snider NT (2018). Vimentin on the move: New developments in cell migration [version 1; referees: 2 approved]. F1000Research.

[56] Pattabiraman S, Azad GK, Amen T (2020). Vimentin protects differentiating stem cells from stress. Sci Rep.

[57] Etienne-Manneville S (2018). Cytoplasmic Intermediate Filaments in Cell Biology. Annu Rev Cell Dev Biol.

[58] Ivaska J, Pallari H-M, Nevo J, Eriksson JE (2007). Novel functions of vimentin in cell adhesion, migration, and signaling. Exp Cell Res.

[59] Jones JCR, Kam CY, Harmon RM, Woychek A V, Hopkinson SB, Green KJ (2017). Intermediate filaments and the plasma membrane. Cold Spring Harb Perspect Biol.

[60] Kalluri R (2009). EMT: when epithelial cells decide to become mesenchymal-like cells. J Clin Invest.

[61] Ivaska J (2011). Vimentin: Central hub in EMT induction?. Small GTPases.

[62] Dustin D, Hall BM, Annapragada A, Pautler RG (2016). Neuroimaging in Alzheimer's disease: preclinical challenges toward clinical efficacy. Transl Res.

[63] Liddelow SA (2015). Development of the choroid plexus and blood-CSF barrier. Front Neurosci.

[64] Hubert V, Chauveau F, Dumot C (2019). Clinical imaging of choroid plexus in health and in brain disorders: a mini-review. Front Mol Neurosci.

[65] Keep RF, Jones HC (1990). A morphometric study on the development of the lateral ventricle choroid plexus, choroid plexus capillaries and ventricular ependyma in the rat. Dev Brain Res.

[66] Choi JD, Moon Y, Kim H-J, Yim Y, Lee S, Moon W-J (2022). Choroid plexus volume and permeability at brain MRI within the Alzheimer disease clinical spectrum. Radiology.

[67] Alisch JSR, Kiely M, Triebswetter C (2021). Characterization of Age-Related Differences in the Human Choroid Plexus Volume, Microstructural Integrity, and Blood Perfusion Using Multiparameter Magnetic Resonance Imaging. Front Aging Neurosci.

[68] Tanifum EA, Starosolski ZA, Fowler SW, Jankowsky JL, Annapragada A V (2014). Cerebral vascular leak in a mouse model of amyloid neuropathology. J Cereb Blood Flow Metab.

[69] Tanifum EA, Dasgupta I, Srivastava M (2012). Intravenous delivery of targeted liposomes to amyloid-β pathology in APP/PSEN1 transgenic mice. PLoS One.

[70] Tanifum EA, Ghaghada K, Vollert C, Head E, Eriksen JL, Annapragada A (2016). A novel liposomal nanoparticle for the imaging of amyloid plaque by magnetic resonance imaging. J Alzheimer's Dis.

[71] Takeuchi H, Iba M, Inoue H (2011). P301S mutant human tau transgenic mice manifest early symptoms of human tauopathies with dementia and altered sensorimotor gating. PLoS One.

[72] Yoshiyama Y, Higuchi M, Zhang B (2007). Synapse loss and microglial activation precede tangles in a P301S tauopathy mouse model. Neuron.

[73] Holmes BB, Furman JL, Mahan TE (2014). Proteopathic tau seeding predicts tauopathy *in vivo*. Proc Natl Acad Sci.

[74] Bargagna-Mohan P, Deokule SP, Thompson K (2013). Withaferin A effectively targets soluble vimentin in the glaucoma filtration surgical model of fibrosis. PLoS One.

[75] Radde R, Bolmont T, Kaeser SA (2006). Aβ42-driven cerebral amyloidosis in transgenic mice reveals early and robust pathology. EMBO Rep.

[76] Christine S-P, Dorothee A, Mairead D (1997). Two amyloid precursor protein transgenic mouse models with Alzheimer disease-like pathology. Proc Natl Acad Sci.

[77] Kurt MA, Davies DC, Kidd M, Duff K, Howlett DR (2003). Hyperphosphorylated tau and paired helical filament-like structures in the brains of mice carrying mutant amyloid precursor protein and mutant presenilin-1 transgenes. Neurobiol Dis.

[78] Sun H, Liu M, Sun T (2019). Age-related changes in hippocampal AD pathology, actin remodeling proteins and spatial memory behavior of male APP/PS1 mice. Behav Brain Res.

[79] Samura E, Shoji M, Kawarabayashi T (2006). Enhanced accumulation of tau in doubly transgenic mice expressing mutant βAPP and presenilin-1. Brain Res.

[80] Hameed S, Fuh J-L, Senanarong V (2020). Role of Fluid Biomarkers and PET Imaging in Early Diagnosis and its Clinical Implication in the Management of Alzheimer's Disease. J Alzheimer's Dis Reports.

[81] Leuzy A, Chiotis K, Lemoine L (2019). Tau PET imaging in neurodegenerative tauopathies—still a challenge. Mol Psychiatry.

[82] Lemoine L, Gillberg P-G, Svedberg M (2017). Comparative binding properties of the tau PET tracers THK5117, THK5351, PBB3, and T807 in postmortem Alzheimer brains. Alzheimers Res Ther.

[83] Baker SL, Harrison TM, Maass A, La Joie R, Jagust WJ (2019). Effect of off-target binding on 18F-Flortaucipir variability in healthy controls across the life span. J Nucl Med.

[84] Volk DE, Lokesh GLR (2017). Development of phosphorothioate DNA and DNA thioaptamers. Biomedicines.

[85] Mu Q, Annapragada A, Srivastava M (2016). Conjugate-SELEX: A high-throughput screening of thioaptamer-liposomal nanoparticle conjugates for targeted intracellular delivery of anticancer drugs. Mol Ther Nucleic Acids.

