# Supplementary material for: A surrogate marker for very early-stage tau pathology is detectable by molecular magnetic resonance imaging
Source: Theranostics. 2022 Jul 18;12(12):5504–21. doi: 10.7150/thno.72258 (PMC9330526; doi:10.7150/thno.72258)
Supplement: Supplementary file 1 — Supplementary figures. [file thnov12p5504s1.pdf]

A surrogate marker for very early-stage tau pathology is detectable by molecular magnetic resonance imaging

Parag Parekh[1+], Qingshan Mu[1+,] Andrew Badachhape[1+], Rohan Bhavane[2], Mayank Srivastava[2], Laxman Devkota[1], Xianwei Sun[1], Prajwal Bhandari[1], Jason L. Eriksen[3], Eric Tanifum[4], Ketan Ghaghada[4], and Ananth Annapragada[4,*]

[1]Baylor College of Medicine, Houston, TX, USA, [2]Texas Children's Hospital, Houston, TX, USA, [3] College of Pharmacy, University of Houston, Houston, TX, USA, [4]Texas Children's Hospital/Baylor College of Medicine, Houston, TX, USA

+: Contributed equally to this manuscript

*: To whom correspondence should be addressed at: annaprag@bcm.edu

SUPPLEMENTARY INFORMATION

Supplementary Table S1

T1 emPAI	Target ID Aptamer Tau1
6.32	60 kDa heat shock protein, mitochondrial OS=Homo sapiens GN=HSPD1 PE=1 SV=2
3.49	Keratin, type II cytoskeletal 1 OS=Homo sapiens GN=KRT1 PE=1 SV=6
1.29	Keratin, type II cytoskeletal 2 epidermal OS=Homo sapiens GN=KRT2 PE=1 SV=2
0.38	Keratin, type II cytoskeletal 6B OS=Homo sapiens GN=KRT6B PE=1 SV=5
0.38	Keratin, type II cytoskeletal 6A OS=Homo sapiens GN=KRT6A PE=1 SV=3
0.11	Keratin, type II cytoskeletal 5 OS=Homo sapiens GN=KRT5 PE=1 SV=3
4.67	Keratin, type I cytoskeletal 10 OS=Homo sapiens GN=KRT10 PE=1 SV=6
1.66	Keratin, type I cytoskeletal 9 OS=Homo sapiens GN=KRT9 PE=1 SV=3
0.97	Keratin, type I cytoskeletal 14 OS=Homo sapiens GN=KRT14 PE=1 SV=4
0.69	Keratin, type I cytoskeletal 17 OS=Homo sapiens GN=KRT17 PE=1 SV=2
5.01	Single-stranded DNA-binding protein, mitochondrial OS=Homo sapiens GN=SSBP1 PE=1 SV=1
2.35	Actin, cytoplasmic 1 OS=Homo sapiens GN=ACTB PE=1 SV=1
0.13	Vimentin OS=Homo sapiens GN=VIM PE=1 SV=4
3.6	40S ribosomal protein S5 (Fragment) OS=Homo sapiens GN=RPS5 PE=1 SV=1
0.2	Microtubule-associated protein 1B OS=Homo sapiens GN=MAP1B PE=1 SV=2
1.27	Elongation factor 1-alpha 1 OS=Homo sapiens GN=EEF1A1 PE=1 SV=1
1.07	Cofilin 1 (Non-muscle), isoform CRA_a OS=Homo sapiens GN=CFL1 PE=1 SV=1
0.28	Leucine-rich PPR motif-containing protein, mitochondrial OS=Homo sapiens GN=LRPPRC PE=1 SV=3
2.74	Peptidyl-prolyl cis-trans isomerase B OS=Homo sapiens GN=PPIB PE=1 SV=2
2.35	Alpha-enolase OS=Homo sapiens GN=ENO1 PE=1 SV=2
0.61	Stress-70 protein, mitochondrial OS=Homo sapiens GN=HSPA9 PE=1 SV=2
1.91	ATP synthase subunit O, mitochondrial OS=Homo sapiens GN=ATP5O PE=1 SV=1
0.73	Serpin H1 OS=Homo sapiens GN=SERPINH1 PE=1 SV=2
0.53	ATP synthase subunit alpha, mitochondrial OS=Homo sapiens GN=ATP5A1 PE=1 SV=1
0.61	ATP synthase subunit gamma, mitochondrial OS=Homo sapiens GN=ATP5C1 PE=1 SV=1
0.94	Tubulin beta chain OS=Homo sapiens GN=TUBB PE=1 SV=1
0.97	Nascent polypeptide-associated complex subunit alpha (Fragment) OS=Homo sapiens GN=NACA PE=1 SV=1
0.25	78 kDa glucose-regulated protein OS=Homo sapiens GN=HSPA5 PE=1 SV=2

T3 emPAI	Target ID Aptamer Tau3
4.45	Keratin, type II cytoskeletal 1 OS=Homo sapiens GN=KRT1 PE=1 SV=6
2.92	Keratin, type II cytoskeletal 2 epidermal OS=Homo sapiens GN=KRT2 PE=1 SV=2
2.7	Vimentin OS=Homo sapiens GN=VIM PE=1 SV=4
0.43	Keratin, type II cytoskeletal 5 OS=Homo sapiens GN=KRT5 PE=1 SV=3
5.32	Keratin, type I cytoskeletal 10 OS=Homo sapiens GN=KRT10 PE=1 SV=6
0.45	Keratin, type I cytoskeletal 14 OS=Homo sapiens GN=KRT14 PE=1 SV=4
1.81	T-cell receptor alpha joining 56 (Fragment) OS=Homo sapiens GN=TRAJ56 PE=4 SV=1
2.62	Actin, cytoplasmic 1 OS=Homo sapiens GN=ACTB PE=1 SV=1
2.35	Actin, cytoplasmic 2 OS=Homo sapiens GN=ACTG1 PE=1 SV=1
0.46	Actin, aortic smooth muscle OS=Homo sapiens GN=ACTA2 PE=1 SV=1
0.37	Serum albumin OS=Homo sapiens GN=ALB PE=1 SV=1
1.16	Keratin, type I cytoskeletal 9 OS=Homo sapiens GN=KRT9 PE=1 SV=3
0.69	60 kDa heat shock protein, mitochondrial OS=Homo sapiens GN=HSPD1 PE=1 SV=2
0.12	Myosin-9 OS=Homo sapiens GN=MYH9 PE=1 SV=4
0.1	Isoform 2 of Myosin-10 OS=Homo sapiens GN=MYH10
0.72	Nascent polypeptide-associated complex subunit alpha (Fragment) OS=Homo sapiens GN=NACA PE=1 SV=1
0.1	Nestin OS=Homo sapiens GN=NES PE=1 SV=2
0.71	Single-stranded DNA-binding protein, mitochondrial OS=Homo sapiens GN=SSBP1 PE=1 SV=1
0.23	40S ribosomal protein S12 OS=Homo sapiens GN=RPS12 PE=1 SV=3
0.7	Serpin H1 (Fragment) OS=Homo sapiens GN=SERPINH1 PE=1 SV=2
0.43	Transcription factor BTF3 homolog 4 OS=Homo sapiens GN=BTF3L4 PE=1 SV=1
0.29	Reticulocalbin-2 OS=Homo sapiens GN=RCN2 PE=1 SV=1
0.26	Prelamin-A/C OS=Homo sapiens GN=LMNA PE=1 SV=1
0.12	THO complex subunit 4 OS=Homo sapiens GN=ALYREF PE=1 SV=1
0.16	Nuclease-sensitive element-binding protein 1 (Fragment) OS=Homo sapiens GN=YBX1 PE=1 SV=1
0.3	Peptidyl-prolyl cis-trans isomerase B OS=Homo sapiens GN=PPIB PE=1 SV=2
0.1	Isoform 2 of Heat shock protein HSP 90-alpha OS=Homo sapiens GN=HSP90AA1
0.12	Heat shock protein HSP 90-beta OS=Homo sapiens GN=HSP90AB1 PE=1 SV=4

Supplementary Table Legend

Supplementary Table S1: Target Identification using aptamer immunoprecipitation and mass spectrometry. Aptamer based immunoprecipitation followed by Mass-Spectrometric analysis of the trypsin digested aptamer-bound complex (LC/MS/MS on LTQ-Orbitrap-XLmass spectrometer with Nanoflex system). The raw data files were processed and searched against the SwissProt_2012_01 (Human) database using the Mascot search engine. Exponentially modified protein abundance index values (emPAI) are reported in the table below.

Supplementary Figure S1

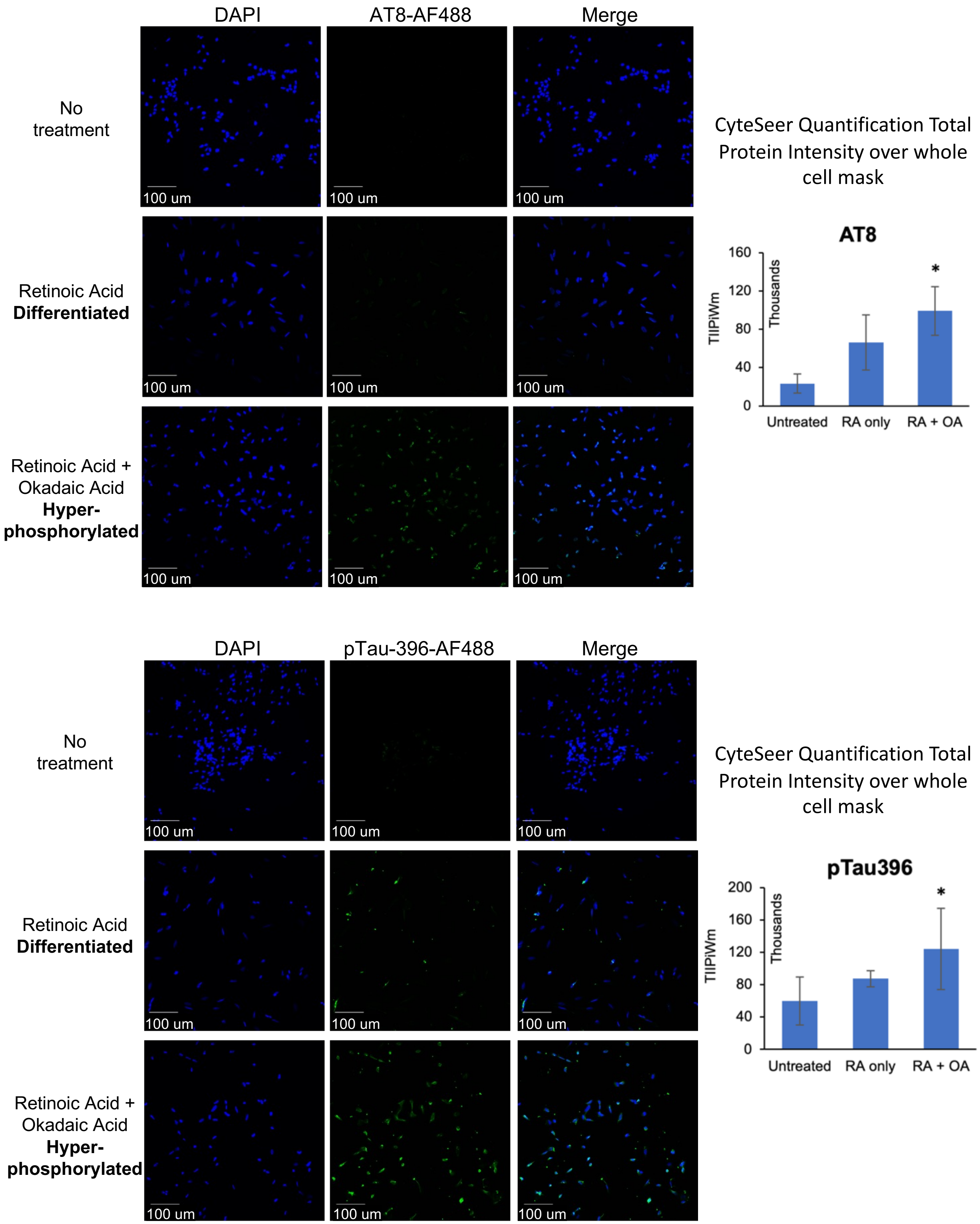

Supplementary Figure S2

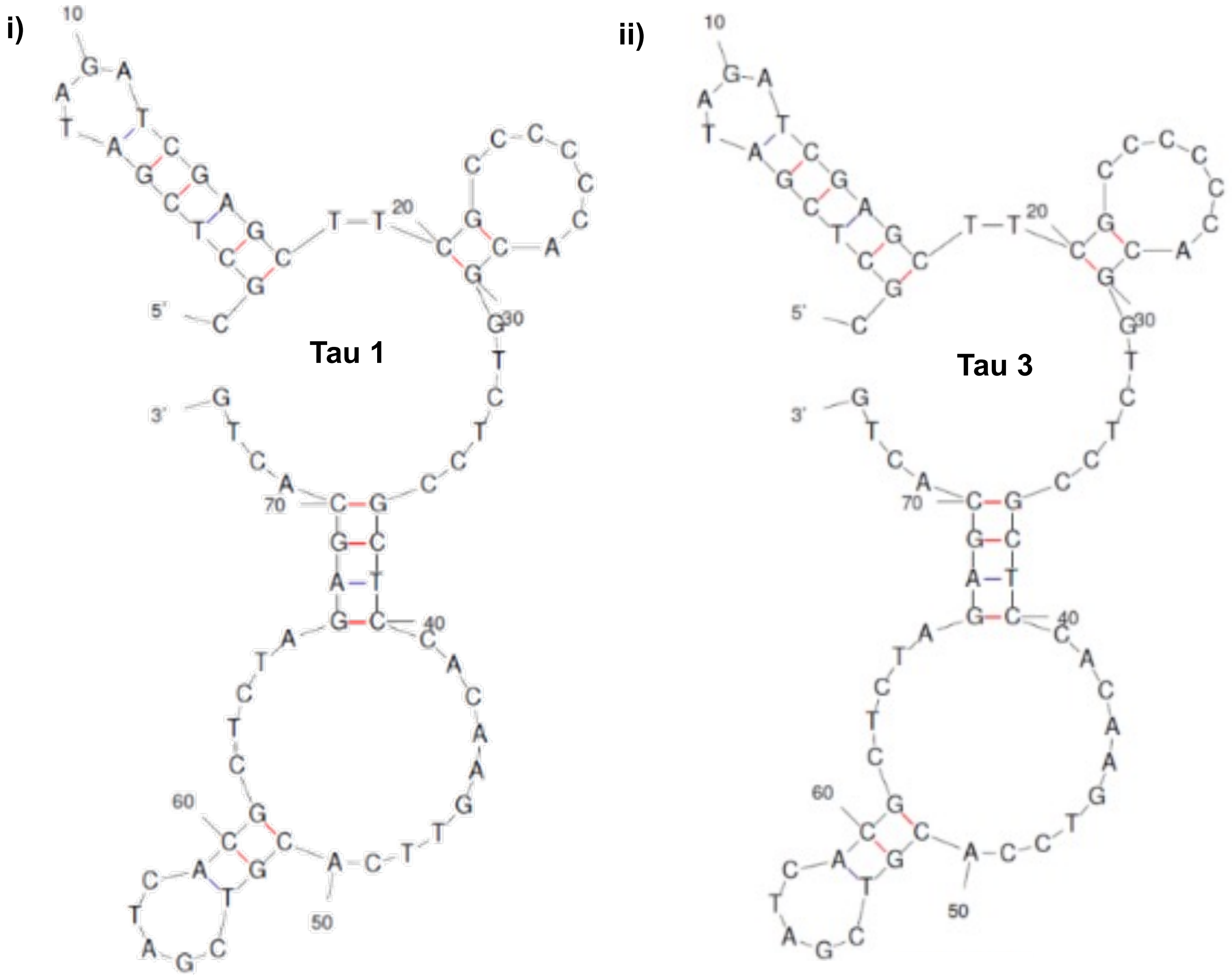

Supplementary Figure S3

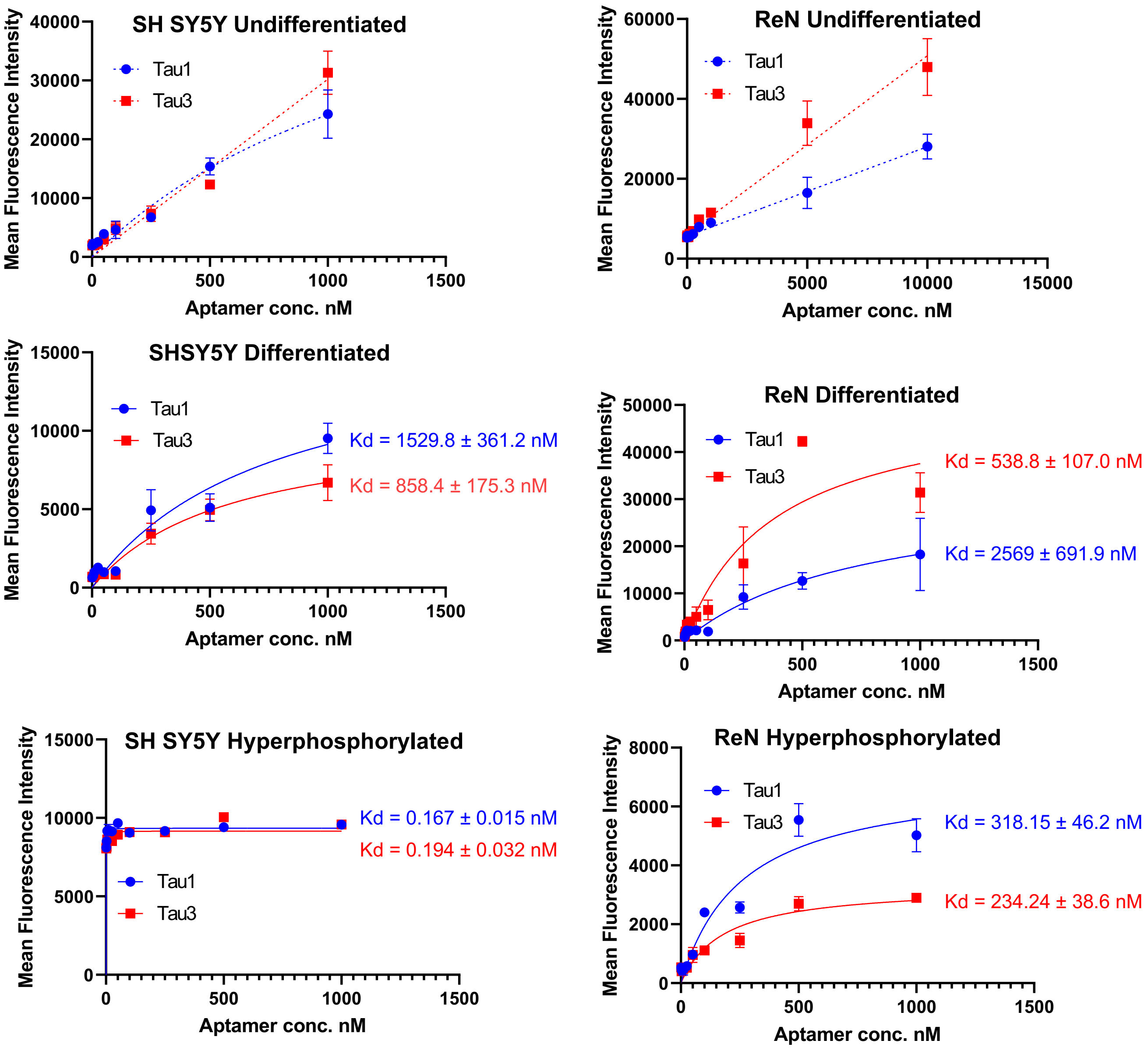

38 **Supplementary Figure S4**

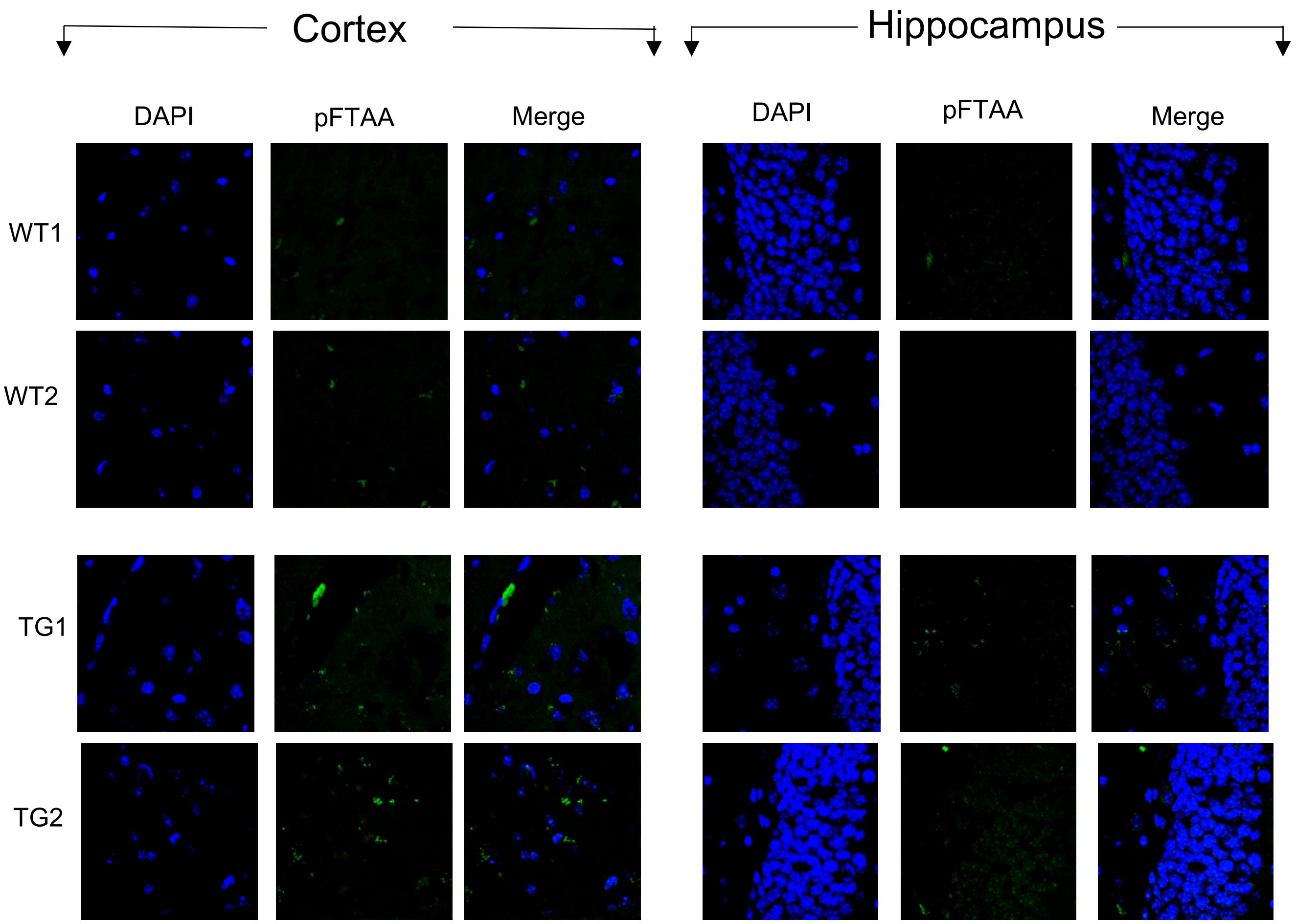

39

40

41

Supplementary Figure S5

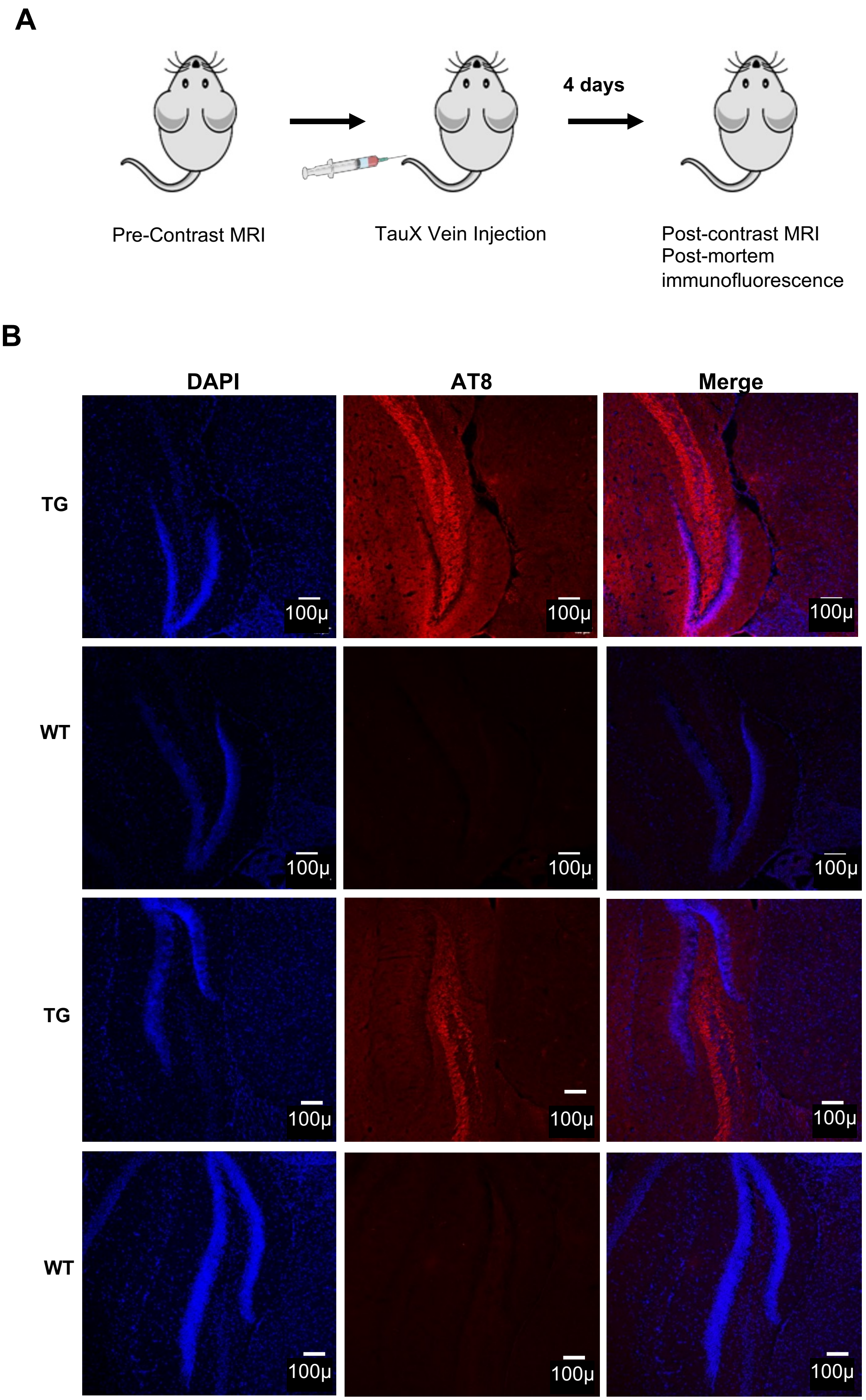

Supplementary Figure S6

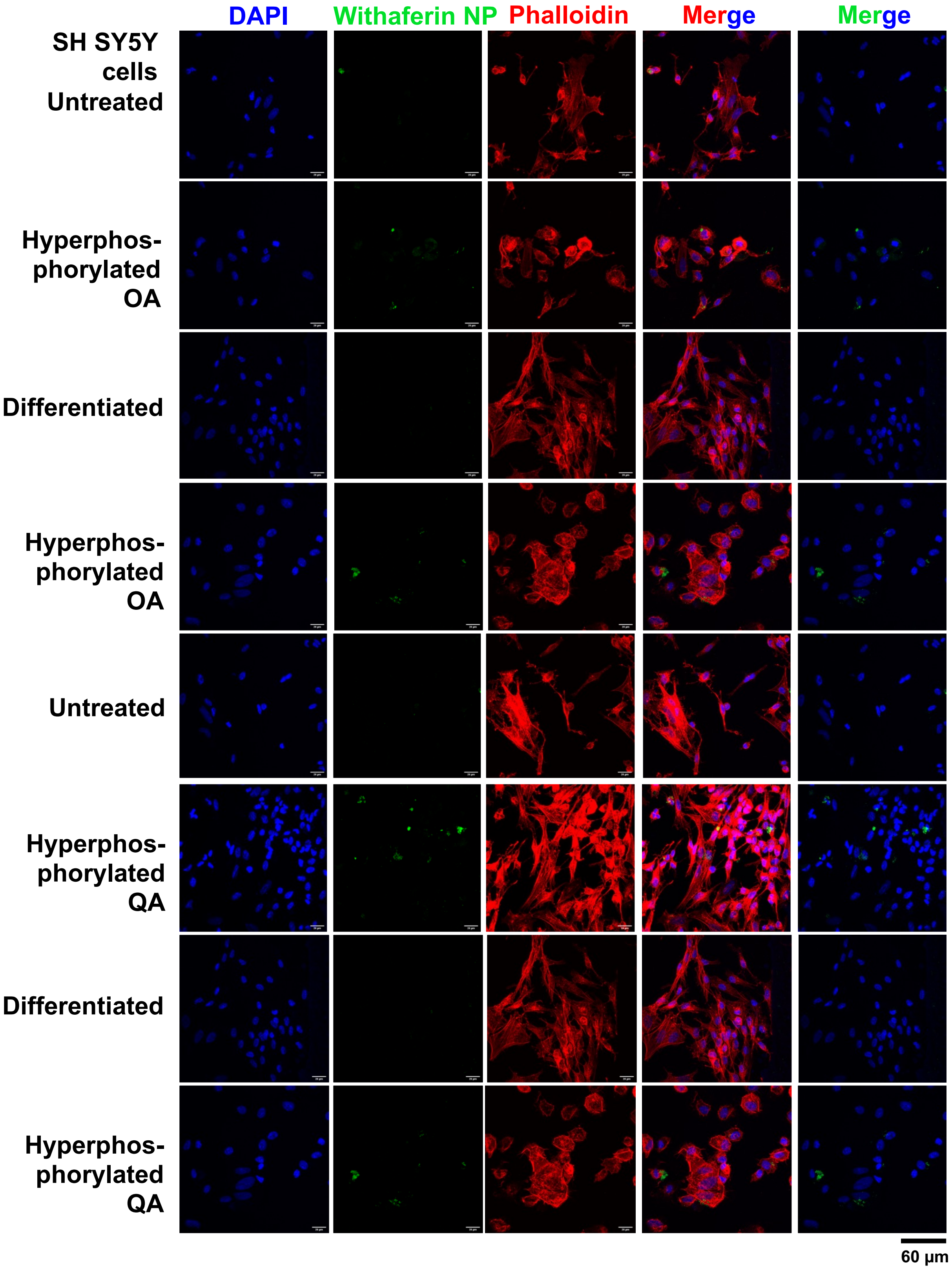

Supplementary Figure S7

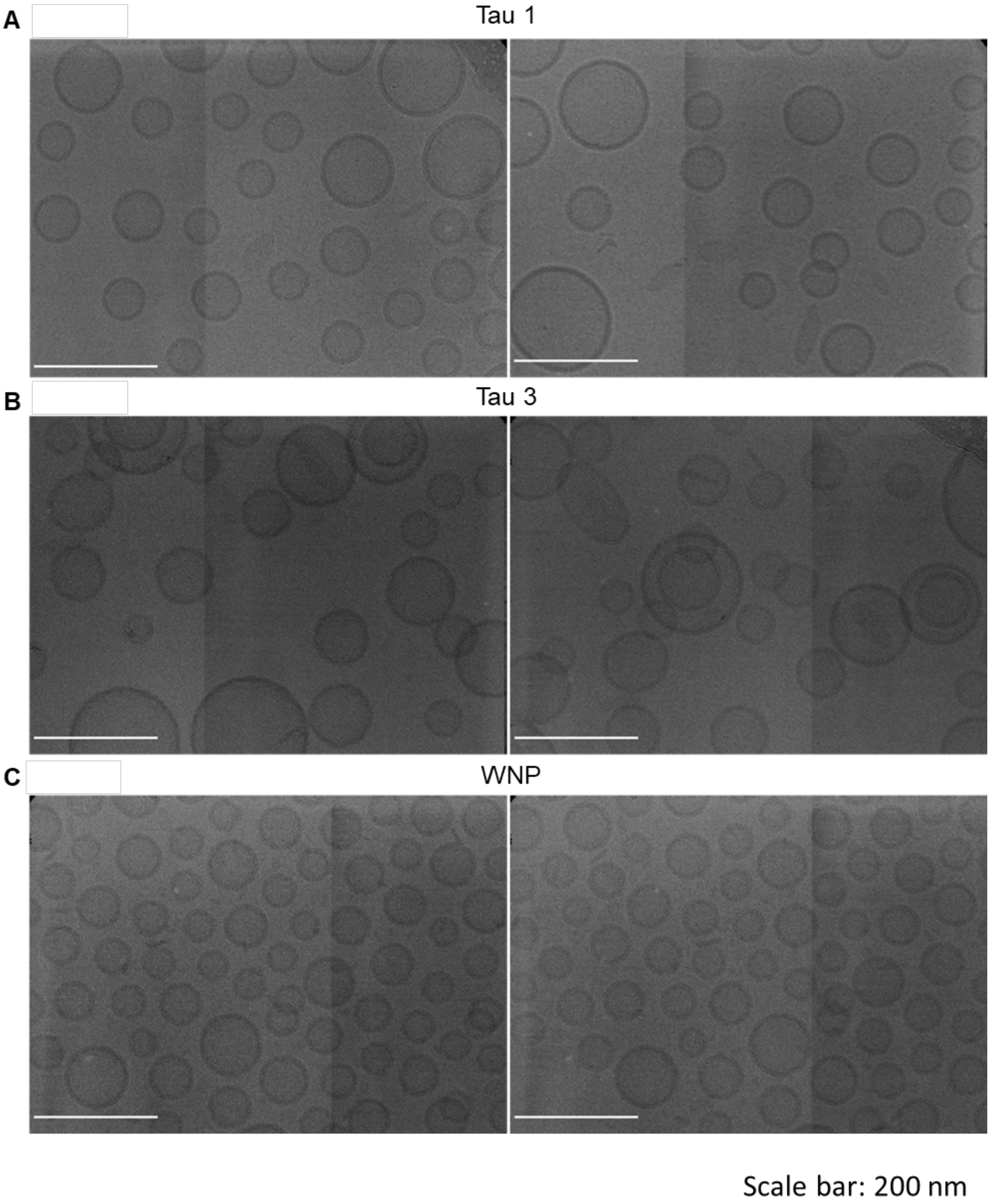

Supplementary Figure S8

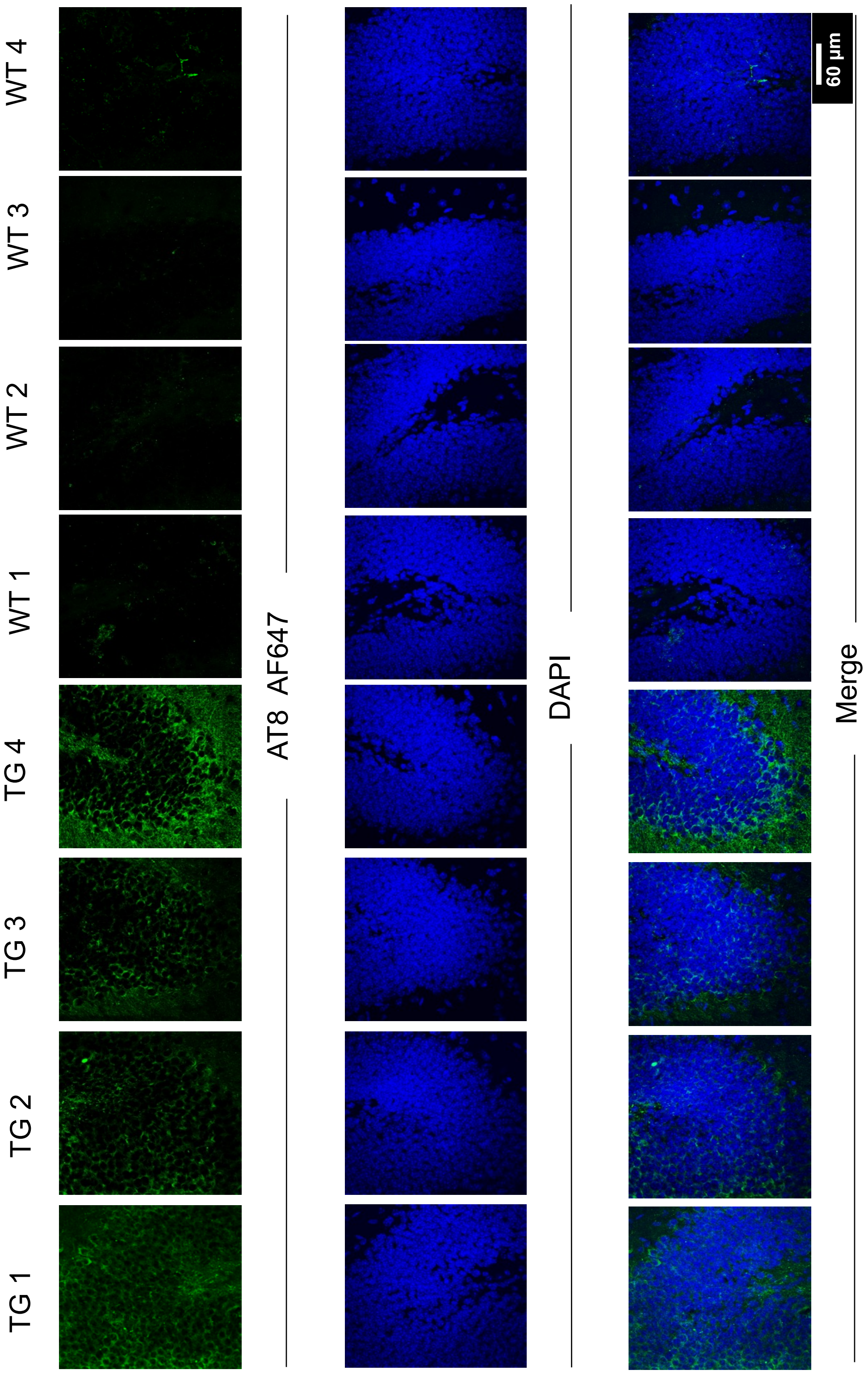

Supplementary Figure S9

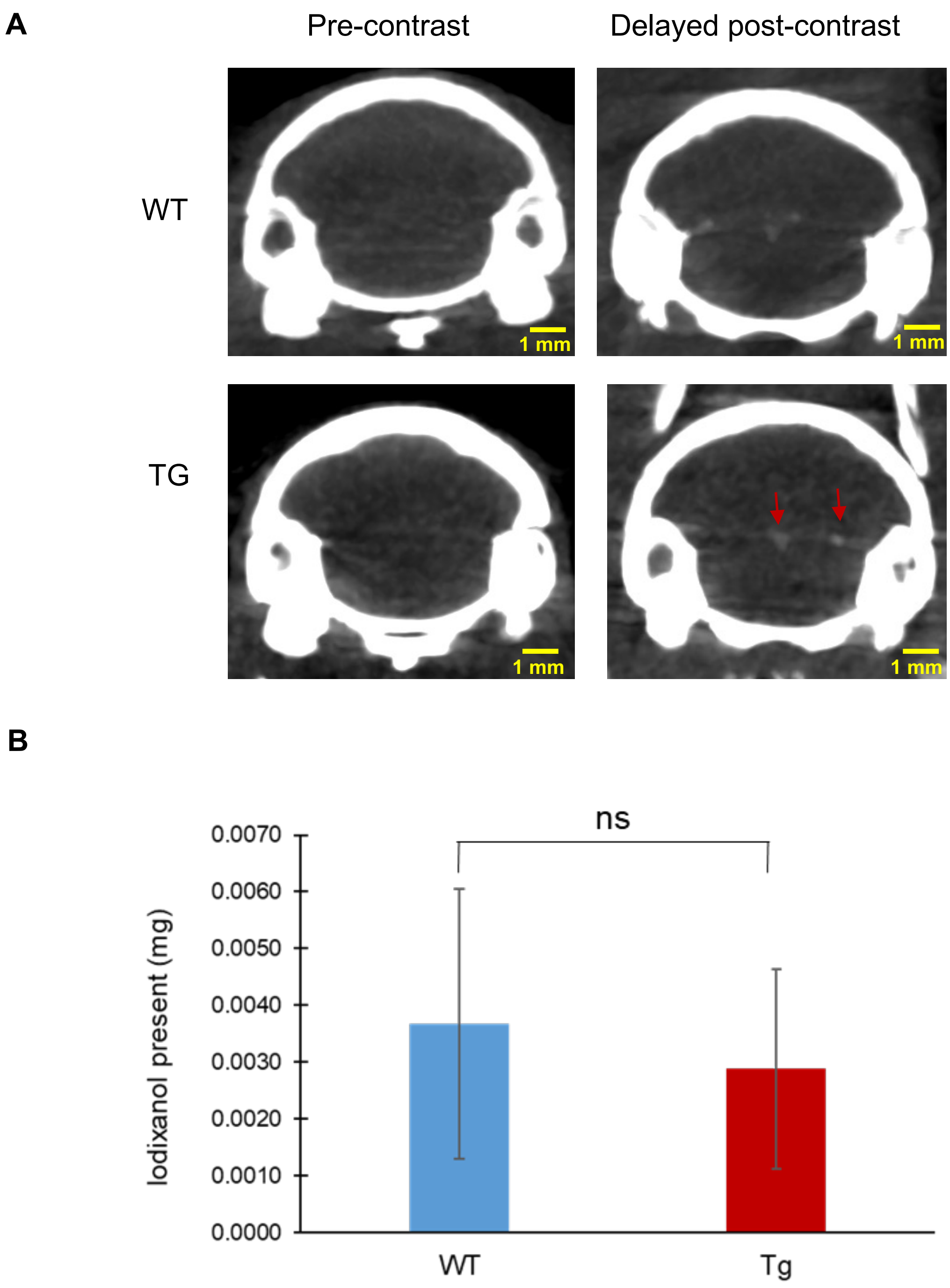

Supplementary Figure S10

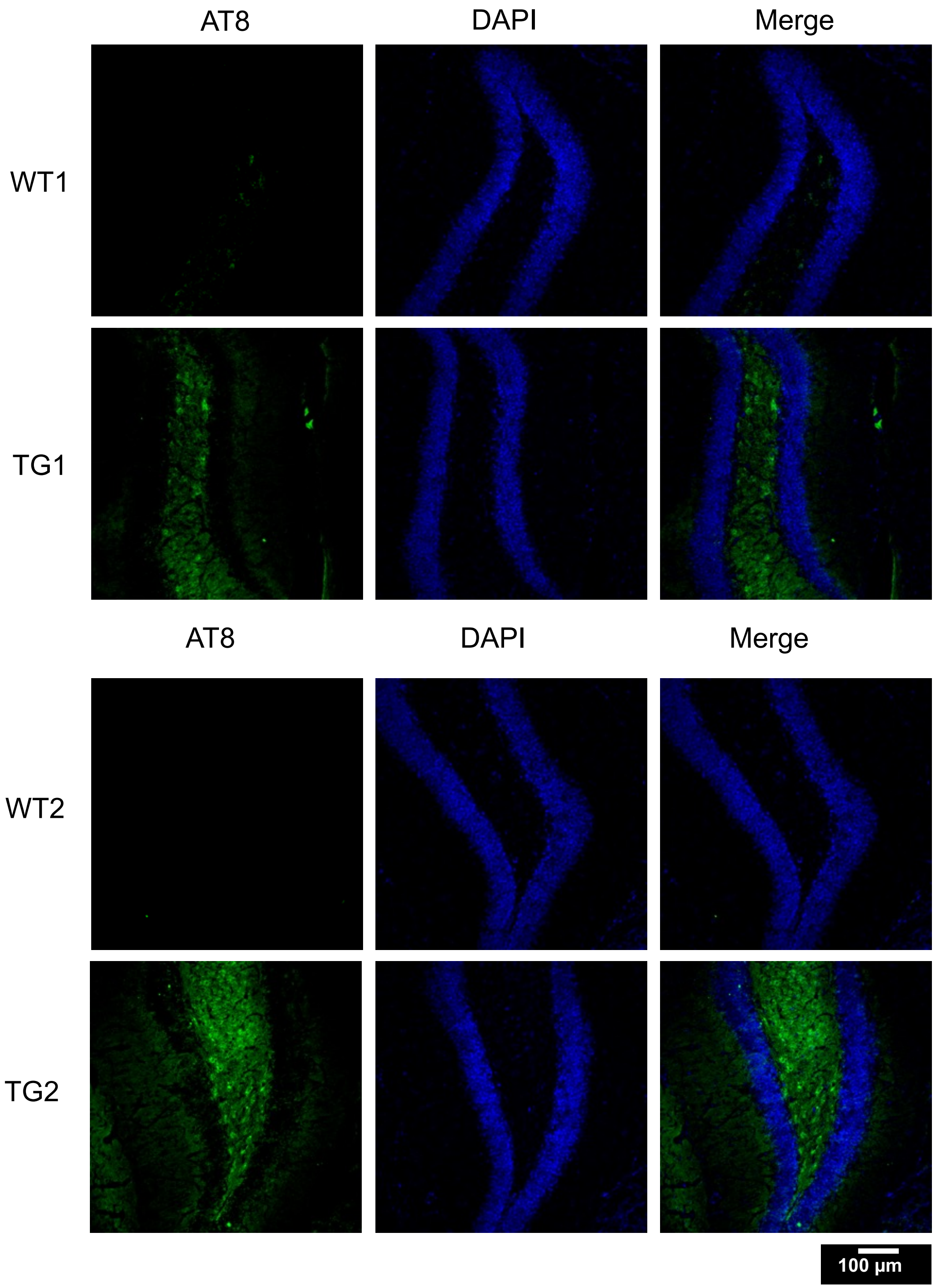

Supplementary Figure S11

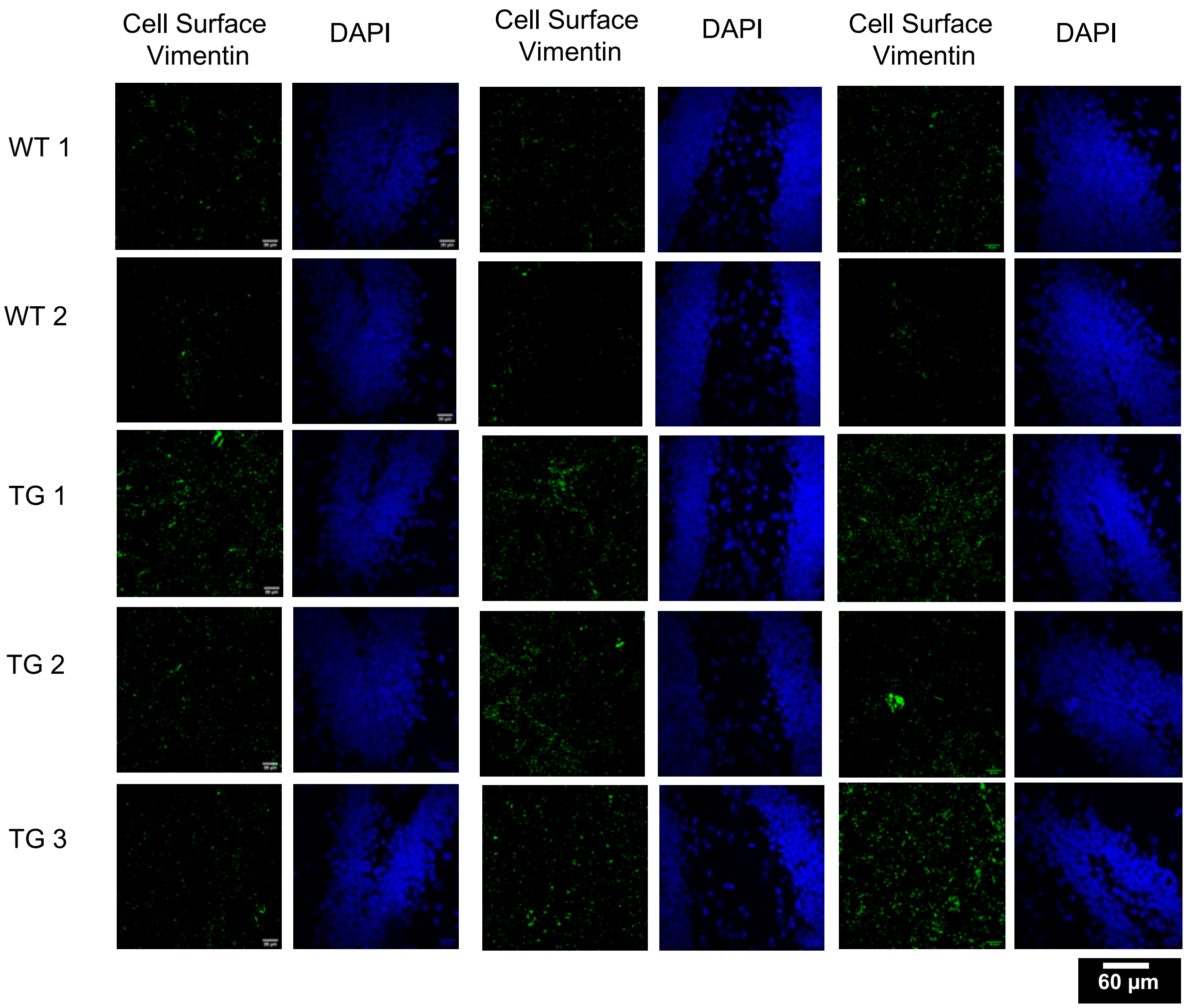

Supplementary Figure Legends

Supplementary Figure S1: Hyperphosphorylative conditions. SHSY5Y cells were differentiated with 30µM Retinoic acid for 10 days. The differentiated cells were treated with 30nM Okadaic acid for 24hrs. Under these conditions Tau is hyperphosphorylated. We detected pTau Thr205/Ser202 (an early phase of phosphorylation) and ptau Ser396 (a late stage of phosphorylation) stained using the antibodies AT8 and 5HCLC respectively. Quantification of the protein expression was conducted by using the automated image analysis software software CyteSeer that reports the total integrated protein intensity over the whole cell mask (TIIPiWm). A minimum of 5 different ROI's with at least 50 cells were analysed. Each bar represents mean of means with SD. * $p<0.05$ compared to untreated control.

Supplementary Figure S2: i) Tau 1 and ii) Tau 3 secondary structure using Mfold.

Supplementary Figure S3: Apparent dissociation constants of Aptamer Tau1 and Tau3Target. SH SY5Y and ReN cells were grown in 96-well plates, Retinoic acid (30µM) was used for differentiation for 10 days. Okadaic acid (30nM) for SH SY5Y and Quinolinic acid (100nM) for ReN cells was used for 24 hr to generate hyperphosphorylative conditions. Saturation binding curves were generated using Cy5 labelled Tau1 and Tau3 using a microplate fluorescent reader. The apparent binding constant Kd was calculated by non-linear regression fitting the equation $Y=Bmax*X/(Kd + X)$. ** error bars smaller than data symbol are not visualized*

Supplementary Figure S4: Labeling of fibrillary Tau inclusions by pFTAA.
P301S mice were aged to 8 months. The development of fibrillary tau filaments was visualized by using pentameric formyl thiophene acetic acid pFTAA that binds fibrillary tau filaments. Frozen mouse brain 30 µm tissue sections were washed with deionized water, equilibrated with 1mg/ml sodium borohydride for 20 min then PBS for 10 min. Sections were incubated with 3 µM pFTAA for 30 min at room temperature and washed 3x with PBS. Staining was found in hippocampus, cortex and hind brain regions consistent with the presence of fibrillary tau filaments.

Supplementary Figure S5: (A) Two-month-old mice were injected with 0.17 mmol Gd/kg dose TauX. Pre- and post-contrast images on day 4 **(B)** Immunofluorescence studies on sections of brains harvested post MRI scans of P301S mice treated with TauX nanoparticles. Brains were perfused with heparin-PBS and fixed in OCT. IF with antibody AT8 probed for presence of ptau species provided high concordance with genotype confirming the presence of hyperphosphorylative conditions in the mice. Images show the Hippocampal region of brain sections.

Supplementary Figure S6: In-vitro binding study of Withaferin nanoparticles. Differentiated and hyperphosphorylative SH SY5Y cells were incubated with Rhodamine labeled Withaferin nanoparticles for 30 min at 37 ºC Binding is specifically observed to cells under hyperphosphorylative conditions.

Supplementary Figure S7: Visualizing liposomal nanoparticle formulations using Cryo-EM. A) Tau1**, B)** Tau 3 and **C)** WNP**.**

Supplementary Figure S8: Phosphorylation status of tau in WNP treated P301S mice.
P301S mice aged 2 months treated with Withaferin A nanoparticles were sacrificed immediately after MRI image acquisition and intact brains were recovered for immunofluorescence. Frozen brain tissue sections were stained with AT8 antibody and the hippocampal region depicted in the images below. Positive signal on TG mice sections but not on the WT confirm the presence of phosphorylated tau in TG mice.

Supplementary Figure S9: CT imaging demonstrate leakage of liposomal nanoparticles in P301S mice.
(A) Axial brain CT images demonstrating leakage of nanoparticles (red arrows) in wild type (WT) and transgenic (Tg) P301S mice. Leakage of nanoparticle CT contrast agent was predominantly observed along the dorsomedial cerebellar artery in the choroid plexus. Studies were performed in 2-3 months old mice. **(B)** Quantification of nanoparticle leakage detected around the dorsomedial cerebellar artery in choroid plexus region in wild type (WT) and transgenic (Tg) P301S mice. The amount of nanoparticle leakage was estimated from CT images acquired using Lip-I agent and expressed as mg iodixanol (active ingredient in Lip-I nanoparticle contrast agent). Studies were performed in 2 months old mice (n=3/genotype). ns: not significant.

Supplementary Figure S10: Evidence of hyperphosphorylation in 2 months old APP/PSEN1 mice.
APP/PSEN1 mice aged 2 months were perfused with heparin/PBS and formalin and brains were isolated and processed to immunolabel frozen sections with AT8 antibody that recognizes and specifically stains phosphorylated tau (Ser202,Thr 205). Transgenic mice showed a higher expression in comparison with WT mice

Supplementary Figure S11: Cell surface vimentin expression on 2 months old P301S mice.
P301S mice aged 2 months were perfused with heparin/PBS and formalin and brains were isolated and processed to immunolabel frozen sections with cell-surface vimentin antibody (clone 84-1, Abnova, 3 fields of view in the hippocampus areas per section) that specifically stains vimentin translocated to the cell surface. Transgenic mice showed a higher expression in comparison with WT mice.
